# Regulation of chromatin accessibility by hypoxia and HIF

**DOI:** 10.1042/BCJ20220008

**Published:** 2022-03-28

**Authors:** Michael Batie, Julianty Frost, Dilem Shakir, Sonia Rocha

**Affiliations:** Department of Molecular Physiology and Cell Signalling, Institute of Systems Molecular and Integrative Biology, University of Liverpool, Crown Street, Liverpool L697ZB, U.K.

**Keywords:** ATAC-seq, chromatin, hypoxia, hypoxia inducible factors, JmjC-histone demethylases, transcription

## Abstract

Reduced oxygen availability (hypoxia) can act as a signalling cue in physiological processes such as development, but also in pathological conditions such as cancer or ischaemic disease. As such, understanding how cells and organisms respond to hypoxia is of great importance. The family of transcription factors called Hypoxia Inducible Factors (HIFs) co-ordinate a transcriptional programme required for survival and adaptation to hypoxia. However, the effects of HIF on chromatin accessibility are currently unclear. Here, using genome wide mapping of chromatin accessibility via ATAC-seq, we find hypoxia induces loci specific changes in chromatin accessibility are enriched at a subset hypoxia transcriptionally responsive genes, agreeing with previous data using other models. We show for the first time that hypoxia inducible changes in chromatin accessibility across the genome are predominantly HIF dependent, rapidly reversible upon reoxygenation and partially mimicked by HIF-α stabilisation independent of molecular dioxygenase inhibition. This work demonstrates that HIF is central to chromatin accessibility alterations in hypoxia, and has implications for our understanding of gene expression regulation by hypoxia and HIF.

## Introduction

Molecular oxygen utilisation and sensing is an essential feature of metazoan life [2]. Decreased oxygen availability (hypoxia) triggers a cellular response, central to which is the activation of transcriptional changes mediated by Hypoxia Inducible Family (HIF) transcription factors [3–6]. HIF heterodimers, typically consisting of an oxygen labile α subunit (HIF-1α and HIF-2α), and a constitutively expressed β subunit (HIF-1β), bind DNA at hypoxia response elements (HREs), and typically function as gene transactivators [7,8]. Canonical regulation of HIF occurs via the Prolyl Hydroxylases (PHD)/von Hippel–Lindau (VHL)/HIF axis. Under normal oxygen tensions, PHDs, a group of 2-OG dependent dioxygenases (2-OGDDs), proline hydroxylate HIF-1α and HIF-2α, targeting them for polyubiquitination by VHL E3 ubiquitin ligase complex and subsequent proteasomal degradation [2,9]. Impairment of PHDs activity in hypoxia, due to their oxygen dependence, stabilises HIF-α subunits and activates the HIF pathway.

At the chromatin level, HIF has been shown to predominantly bind RNA pol2 loaded, accessible chromatin regions with pre-established and primed, promoter enhancer loops [4,6,10,11]. HIF function is mediated by co-activators, including CREB-binding protein (CBP)/p300, SET Domain Containing 1B Histone Lysine Methyltransferase (SET1B), CDK8 and KAT5 [12]. Chromatin also directly senses oxygen through 2-OGDDs [13–15]. Inhibition of certain Ten-eleven Translocation (TET) methylcytosine dioxygenases and Jumonji C (JmjC)-domain containing histone demethylases in hypoxia alters DNA and histone methylation landscape, respectively, and co-ordinates transcriptional responses [13,14,16]. Recently, several studies have used Assay for Transposase-Accessible Chromatin using sequencing (ATAC)-seq to explore the chromatin accessibility landscape in response to oxygen fluctuation [17–20]. These studies reveal that hypoxia induces dynamic changes in chromatin accessibility in cell culture models [17,19,20]. However, the roles of HIF and 2-OGDD oxygen sensing in hypoxia induced chromatin accessibility have not been studied, and remains an important question, as more inhibitors of these pathways are developed to enter the clinical setting.

Here, using ATAC-seq, we have investigated effects of oxygen deprivation and reoxygenation on chromatin accessibility in cells in culture. We also measured transcript changes in hypoxia with RNA-seq and analysed the role of HIF in this process using a specific stabiliser of HIF-α as well as siRNA-mediated depletion of HIF-1β. Integrative analysis of ATAC-seq with RNA-seq reveals that hypoxia induces co-ordinated and specific changes to chromatin accessible regions, which correlate with gene expression changes, agreeing with previously published data in other cell lines [17,20]. Furthermore, most hypoxia inducible changes to chromatin accessibility across the genome are HIF dependent and rapidly reversible upon reoxygenation. Additionally, HIF stabilisation, independent of 2-OGDD inhibition, is sufficient to partially mimic hypoxia-induced changes in chromatin accessibility. Lastly, we find that H3K4me3 levels correlate with accessibility changes in hypoxia and provide evidence for a role of KDM5A in regulation of chromatin accessibility in hypoxia.

## Results

### Genome wide mapping of the chromatin accessibility landscape in normoxia and hypoxia

**Figure 1.**
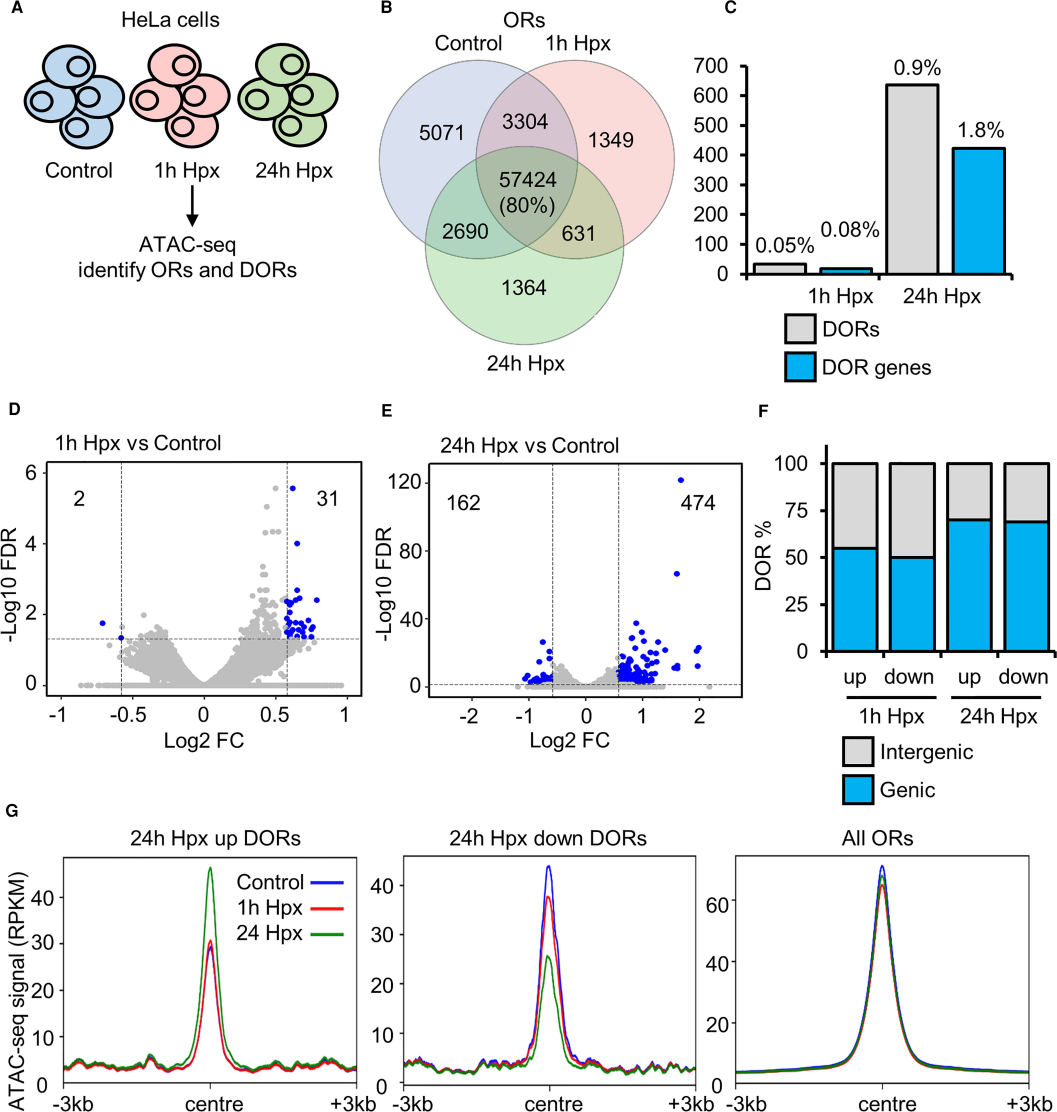
Chromatin accessibility changes in response to hypoxia. (**A**) ATAC-seq (*n* = 2) in HeLa cells cultured at 21% oxygen, transfected with control siRNA and exposed to 0 h (control), 1 h and 24 h 1% oxygen (hypoxia (Hpx)). (**B**) Overlap of open chromatin regions (ORs). (**C**) Number of high stringency (log_2_ fold change >±0.58 and FDR <0.05) differentially open chromatin regions (DORs) and genes with DORs (DOR genes), and percentage relative to total ORs and OR genes. (**D**,**E**) Volcano plots for 1 h hypoxia vs control DOR analysis and 24 h hypoxia vs control DOR analysis, blue points indicate high stringency DORs. (**F**) Genomic location of DORs. (**G**) Metagene plots of ATAC-seq signal (RPKM) at the indicated regions.

The hypoxia response in cells involves a co-ordinated transcriptional programme [7]. However, chromatin accessibility dynamics in response to hypoxia are not well defined. To investigate the effect of acute hypoxia on chromatin accessibility, we performed ATAC-seq in HeLa cells cultured at 21% oxygen (control) or exposed to one and 24 h of hypoxia (1% oxygen) (Figure 1A; Supplementary Dataset S1). Seventy-one thousand six hundred fifty-one high confidence (identified in all biological replicates within a condition, each with an FDR < 1 × 10^−15^) open chromatin regions (ORs) are identified across all time points, with 80% present in all time points (Figure 1B). Twenty-seven thousand nine hundred one OR genes (ORs at genic regions) are identified across all time points, with 91% present in all time points (Supplementary Figure S1A). Data is in concordance with current ENCODE standards for ATAC-seq (Supplementary Dataset S1) and similar regions of open chromatin are identified comparing to other published HeLa ATAC-seq (Supplementary Figure S1B), demonstrating high data quality.

Differential open region (DOR) analysis (Supplementary Dataset S2) identified site specific changes at ORs in response to hypoxia. Thirty-three high stringency (log_2_ fold change >±0.58 and FDR <0.05) DORs are present in response to 1 h hypoxia (Figure 1C,D) and 636 high stringency DORs are present in response to 24 h hypoxia (Figure 1C,E). Eighteen DOR genes and 422 DOR genes are identified in response to one h and 24 h hypoxia, respectively (Figure 1C). Of the one h hypoxia DORs, 31/33 are up-regulated and 2/33 are down-regulated (Figure 1D). When analysing the 24 h hypoxia DORs, 474/636 are up-regulated and 162/636 are down-regulated (Figure 1E). DORs are spread across genic and intergenic regions (Figure 1F). Mapping ATAC-seq signal across hypoxia DORs shows changes in chromatin accessibility induced by hypoxia are loci specific (Figure 1G; Supplementary Figure S1C,D). When using lower stringency DOR analysis (FDR < 0.1) we find 445 DORs and 336 DOR genes in response to one h hypoxia, and 4877 DORs and 2955 DOR genes in response to 24 h hypoxia (Supplementary Figure S1E,F). Interestingly, this lower stringency analysis produced similar number of changes to those identified in HUVEC cells exposed to hypoxia [17].

These results show that hypoxia induces changes in chromatin accessibility at a specific set of loci in HeLa cells, with most changes occurring at later than one h of hypoxia.

### Hypoxia induced changes in chromatin accessibility correlate with changes in gene expression

**Figure 2.**
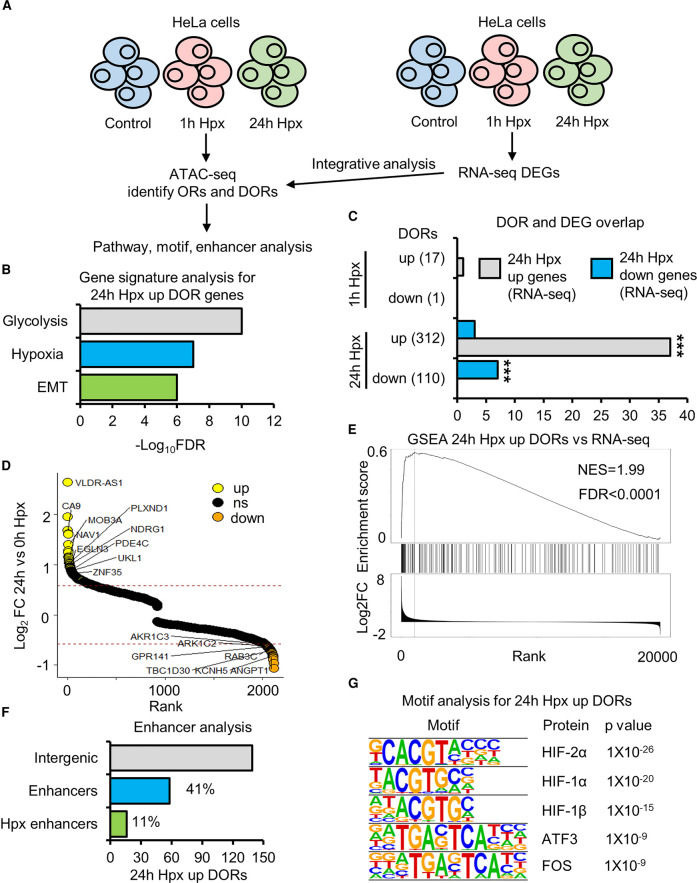
Hypoxia inducible changes in open chromatin are enriched at hypoxia transcriptionally regulated genes. (**A**) ATAC-seq (*n* = 2) and RNA-seq (*n* = 3) in HeLa cells cultured at 21% oxygen, transfected with control siRNA and exposed to 0 h (control), 1 h and 24 h 1% oxygen (hypoxia (Hpx)). (**B**) Gene signature analysis. (**C**) Overlap between genes with differentially open chromatin regions (DOR genes) and genes with differential RNA expression (DEG) in response to hypoxia. Statistical significance was determined via Fisher's exact test, *** *P* < 0.001. (**D**) Gene list ranked from high to low fold change in chromatin accessibility in response to 24 h hypoxia. Up-regulated DOR genes are coloured yellow, down-regulated DOR genes are coloured orange. Some hypoxia up-regulated DEG and DOR genes and down-regulated DEG and DOR genes are labelled. (**E**) GeneSet Enrichment Analysis between 24 h hypoxia up-regulated DOR genes and a list of genes ranked from high to low 24 h hypoxia RNA expression fold change. (**F**) Percentage of 24 h hypoxia up-regulated DORs at intergenic regions that are active enhancers and active enhancers linked to the promoters of genes with 24 h hypoxia up-regulated RNA expression. (**G**) Motif enrichment analysis, top 5 enriched motifs are displayed.

Hypoxia responsive chromatin accessible regions were investigated for the associated gene signatures (Figure 2A). Glycolysis, hypoxia and EMT gene signatures are enriched at 24 h hypoxia up-regulated DOR genes (Figure 2B). No statistically significant pathway enrichment was found for 24 h hypoxia down-regulated DOR genes or one h hypoxia DOR genes. To determine how changes in gene expression correlate with changes in chromatin accessibility, we performed RNA-seq in HeLa cells exposed to zero, one and 24 h of hypoxia (Supplementary Dataset S3). Twenty-five (23 up-regulated, two down-regulated) differentially expressed genes (DEGs) in response to one h hypoxia (Supplementary Dataset S3), and 1330 DEGs (1088 up-regulated, 242 down-regulated) in response to 24 h hypoxia are identified from this analysis (Supplementary Dataset S3). From integrative analysis of ATAC-seq with RNA-seq data, we found a subset of genes with hypoxia induced differential expression, which also possess changes in chromatin accessibility (Figure 2C,D; Supplementary Dataset S4). Twenty-four h hypoxia up-regulated DOR genes show significant correlation with 24 h hypoxia up-regulated expression genes, 37 of the 24 h hypoxia up-regulated DOR genes have increased gene expression (Figure 2C), among these are the well characterised, core hypoxia responsive genes, *CA9, NDRG1* and *EGNL3* (protein name PHD3) (Figure 2D). GeneSet Enrichment Analysis also confirmed these results (Figure 2E). Interestingly, 24 h hypoxia down-regulated OR genes also show significant correlation with 24 h hypoxia down-regulated expression genes (Figure 2C,D).

The aforementioned analysis is specific to genic (promoter and gene body) DORs. To functionally annotate changes in chromatin accessibility at intergenic regions, we performed enhancer analysis and found that 42% of intergenic 24 h hypoxia up-regulated DORs are at active enhancers (Figure 2E). Eleven percent (16/142) are enhancer partners for the promoters of genes whose expression is up-regulated at 24 h hypoxia (Figure 2F). These include the promoters of the well characterised, core hypoxia responsive genes, *SCL2A3* (protein name GLUT3) and *NDRG1*. Thus, changes in accessibility at hypoxia responsive genes occur at both gene proximal and distal regulatory elements.

Lastly, motif enrichment analysis shows HIF subunits motifs are enriched at 24 h hypoxia up-regulated DORs (Figure 2G), suggesting a role of HIF in coordination of changes in chromatin accessibility in hypoxia. The motifs from this analysis are also enriched at basal (normoxic) ORs present on genes with up-regulated expression in hypoxia (Supplementary Figure S2). Therefore, motif analysis does not identify any factors potentially controlling chromatin accessibility in hypoxia, distinct from those controlling gene expression.

### Hypoxia induced changes in chromatin accessibility are mostly sensitive to reoxygenation and are HIF dependent

**Figure 3.**
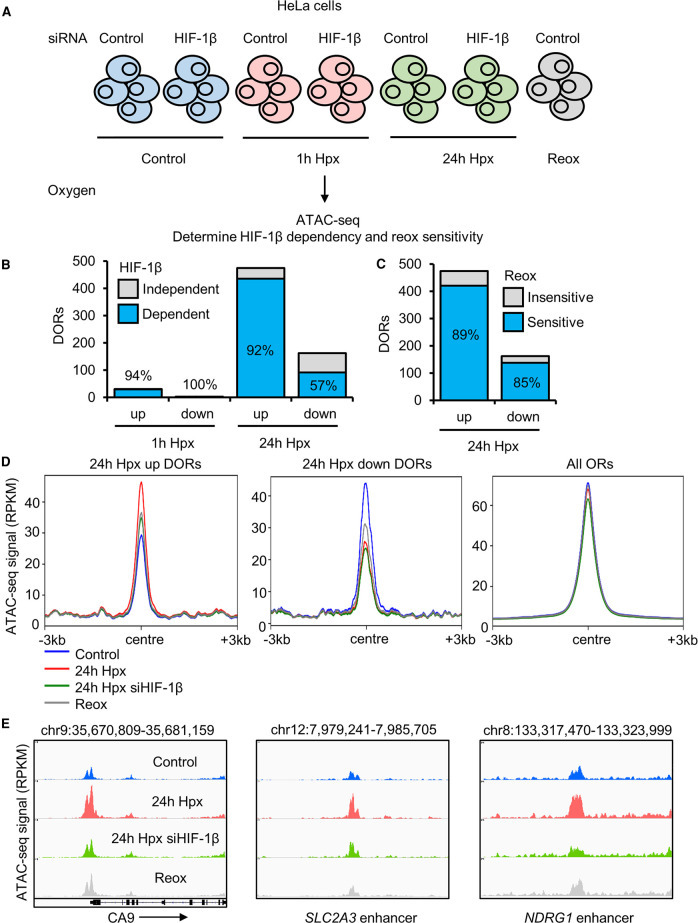
Hypoxia inducible changes in open chromatin are mainly sensitive to reoxygenation HIF dependent. (**A**) ATAC-seq (*n* = 2) in HeLa cells cultured in 21% oxygen; transfected with control siRNA or HIF-1β siRNA, and exposed to 0 h (control), 1 h, 24 h 1% oxygen (hypoxia (Hpx)) and 24 h hypoxia followed by 1 h at 21% oxygen (reoxygenation (Reox)). (**B**) HIF-1β dependence of hypoxia differentially open chromatin regions (DORs), percentage of HIF-1β dependent DORs are labelled. (**C**) Reoxygenation sensitivity of 24 h hypoxia DORs, percentage of reoxygenation sensitive DORs are labelled. (**D**) Metagene plots of ATAC-seq signal (RPKM) at the indicated regions. (**E**) Coverage tracks of ATAC-seq signal at the *CA9* promoter, *SLC2A3* (protein name GLUT3) enhancer and *NDRG1* enhancer.

HIF is the master regulator of transcriptional changes in response to hypoxia; however, its role in chromatin accessibility regulation has been elusive. To ascertain the dependence of HIF on hypoxia inducible changes to chromatin accessibility, we performed ATAC-seq in cells where HIF-1β (the obligate partner for HIF heterodimer complexes) was depleted by siRNA prior to hypoxia exposure (Figure 3A; Supplementary Datasets S1, S2, S5). One h hypoxia DORs were almost exclusively dependent on HIF-1β (Figure 3B; Supplementary Dataset S5). At 24 h hypoxia, requirement for HIF is favoured at sites with increased accessibility over reduced accessibility, with 92% of 24 h hypoxia up-regulated ORs and 57% of 24 h hypoxia down-regulated DORs requiring HIF-1β (Figure 3B; Supplementary Dataset S5). Hypoxia DORs are classed as dependent on HIF-1β if they are they not identified as DORs with HIF-1β siRNA treatment when comparing to control (0 h hypoxia, control siRNA). Validation of siRNA depletion of HIF-1β is confirmed by immunoblotting (Supplementary Figure S3A).

To elucidate the sensitivity of hypoxia induced changes in chromatin accessibility to fluctuations in oxygen levels, we included a reoxygenation condition in our analysis (24 h hypoxia (1% oxygen), followed by 1 h at normoxia (21% oxygen) (Figure 3A; Supplementary Datasets S1, S2, S6). The vast majority of 24 h hypoxia DORs (89% of up-regulated DORs and 85% of down-regulated DORs) return to near normoxic levels upon reoxygenation (Figure 3C; Supplementary Dataset S6). DORs are classed as reoxygenation sensitive if they are not identified as DORs in reoxygenation condition compared with control (0 h hypoxia, control siRNA). As a control for hypoxia and reoxygenation, immublotting of HIF-1α and HIF-2α was performed (Supplementary Figure S3B). HIF-1α increases at 1 h and 24 h hypoxia and this increase is lost with reoxygenation. HIF-2α levels are also increased at 1 h and hypoxia compared with control, levels at 24 h hypoxia are higher than at 1 h hypoxia, and HIF-2α stabilisation is lost with reoxygenation.

PCA analysis shows ATAC-seq sample clustering by treatment (Supplementary Figure S3C). As found with the analysis of hypoxia treatment, reoxygenation and HIF-1β depletion cause loci specific changes as opposed to genome wide changes in chromatin accessibility (Figure 3D; Supplementary Figure S3D,E). Coverage tracks of a subset of hypoxia up-regulated DORs at hypoxia transcriptionally up-regulated gene promoters/enhancers are displayed, demonstrating HIF-1β dependence and reoxygenation sensitivity of hypoxia inducible chromatin accessibility changes.

These data indicate that changes in chromatin accessibility in hypoxia are highly dependent on HIF, particularly regards to loci with increased accessibility in hypoxia. In addition, hypoxia-induced chromatin changes are dependent on oxygen availability and rapidly reversed upon reoxygenation.

### VH298 mediated HIF stabilisation is sufficient to induce changes to chromatin accessibility

**Figure 4.**
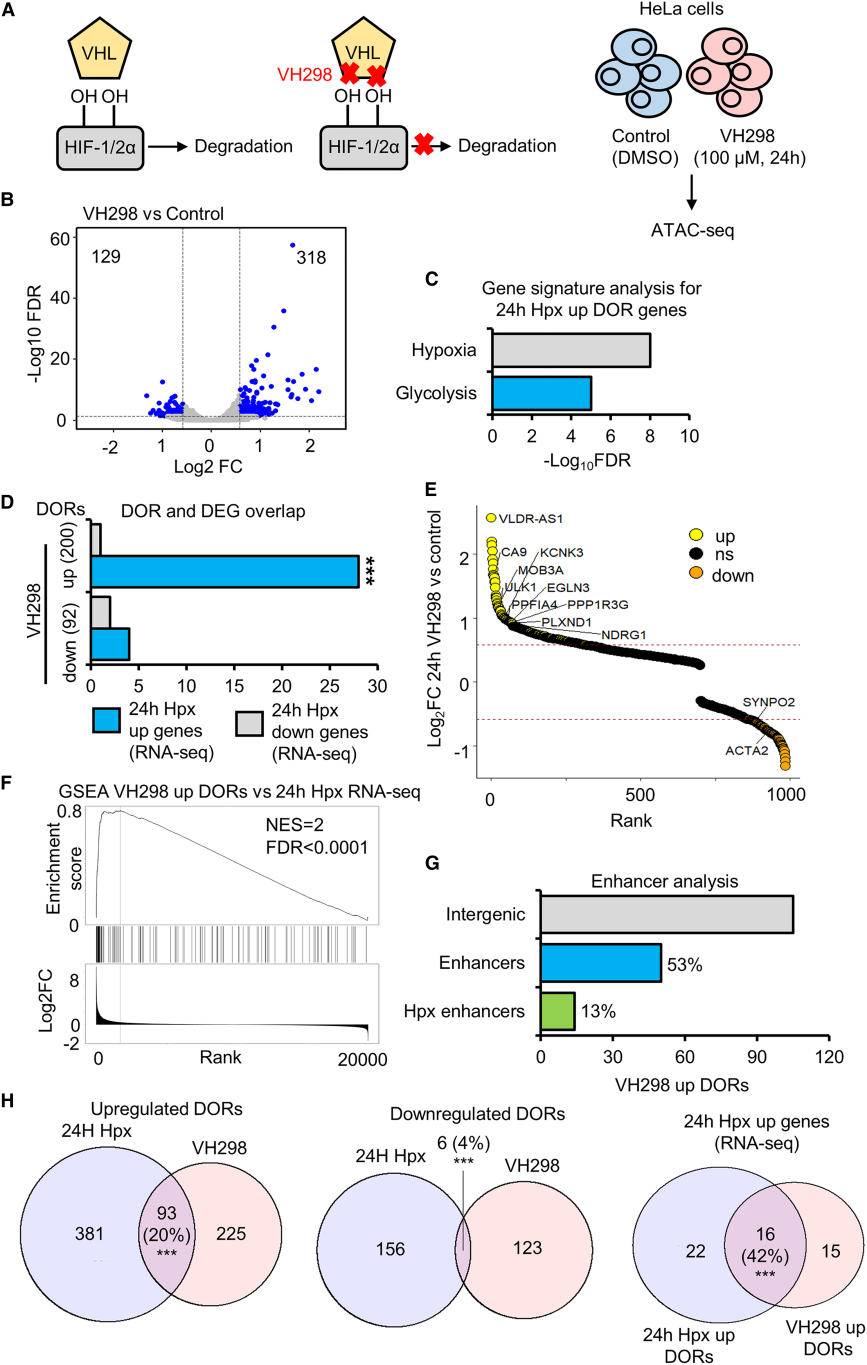
Chromatin accessibility changes in response to HIF stabilisation via VH298. (**A**) ATAC-seq (*n* = 2) in HeLa cells cultured at 21% oxygen and treated with DMSO (control) and 100 µM VH298 for 24 h. (**B**) Volcano plot for differentially open chromatin region (DOR) analysis, blue points indicate high stringency DORs. (**C**) Gene signature analysis. (**D**) Overlap between genes with differentially open chromatin regions (DOR genes) in response to VH298 and genes with differential RNA expression (RNA-seq (*n* = 3)) (DEGs) in response to hypoxia. Statistical significance was determined via Fisher's exact test *** *P* < 0.001. (**E**) Gene list ranked from high to low fold change in chromatin accessibility in response to VH298. Up-regulated DOR genes are coloured yellow, down-regulated DOR genes are coloured orange. Some up-regulated hypoxia DEG and VH298 DOR genes and down-regulated hypoxia DEG and VH298 DOR genes are labelled. (**F**) GeneSet Enrichment Analysis between VH298 up-regulated OR genes and a list of genes ranked from high to low 24 h hypoxia RNA expression fold change. (**G**) Percentage of VH298 up-regulated DORs at intergenic regions that are active enhancers and active enhancers linked to the promoters of genes with 24 h hypoxia up-regulated RNA expression. (**H**) Overlap of VH298 and 24 h hypoxia DORs. Statistical significance was determined via hypergeometric test *** *P* < 0.001.

In addition to HIF stabilisation, 2-OGDD inhibition in hypoxia can alter modifications of other targets, including histones [13,14]. To uncouple the effects of HIF stabilisation and 2-OGDD inhibition on chromatin accessibility, we performed ATAC-seq in HeLa cells treated for 24 h with DMSO (control) and 24 h, 100 µM VH298, a specific chemical inhibitor of the hydroxylated HIF-α binding pocket of VHL [21–23] (Figure 4A; Supplementary Dataset S1). Immunoblot analysis confirmed HIF-1α and HIF-2α stabilisation in response to VH298 treatment (Supplementary Figure S4A). Seventy-two thousand one hundred and thirty-seven high confidence (identified in all biological replicates within a condition, each with an FDR < 1 × 10^−15^) ORs are found across control and VH298 treated samples, with 85% found in both (Supplementary Figure S4B). Twenty-three thousand nine hundred and ninty-three OR genes are present across control and VH298 treated samples, with 93% present in both (Supplementary Figure S4B). VH298 DOR analysis reveals 447 high stringency (log_2_ fold change >±0.58 and FDR <0.05) DORs and 292 DOR genes in response to 24 h VH298 treatment (Supplementary Figure S4C; Supplementary Dataset S2). Of the VH298 DORs, 318/447 are up-regulated and 129/447 are down-regulated (Figure 4B). Sixty-seven percent of up-regulated VH298 DORs and 72% of VH298 down-regulated DORs are at genic regions (Supplementary Figure S4D). As with hypoxia, VH298 induces loci specific changes in chromatin accessibility (Supplementary Figure S4E,F). Lower stringency DOR analysis (FDR <0.1) finds 1555 DORs and 1031 DOR genes in response to VH298 treatment (Supplementary Figure S4G,H).

Gene signature analysis identified hypoxia and glycolysis pathways as enriched at VH298 up-regulated DOR genes (Figure 4C). These signatures were also enriched in the data related to 24 h hypoxia. Similarly, as with 24 h hypoxia exposure, VH298 up-regulated DOR genes have significant overlap with 24 h hypoxia up-regulated expression genes (Figure 4D,E), and are enriched at 24 h hypoxia up-regulated expression genes as determined by GeneSet Enrichment Analysis (Figure 4F). Integrative analysis with the HACER enhancer database shows that 53% of intergenic VH298 up-regulated DORs are at enhancer regions and 13% (14/105) are enhancer partners linked to promoters of 24 h hypoxia up-regulated expression genes (Figure 4G). Motif enrichment analysis reveals HIF subunit-binding motifs are overrepresented in VH298 up-regulated DOR genes (Supplementary Figure S4I). These data show that, HIF stabilisation, independent of dioxygenase inhibition, is sufficient to trigger loci specific changes in chromatin accessibility linked to hypoxia regulated genes.

We next directly compared chromatin accessibility responses between hypoxia and VH298 (Supplementary Dataset S7). Exposure to 24 h hypoxia induced changes in chromatin accessibility at 42% more genomic loci than 24 h VH298 treatment (636 DORs compared with 447 DORs). There is higher similarity of up-regulated responses, with 20% of hypoxia up-regulated sites also up-regulated by VH298 whereas only 4% of hypoxia down-regulated sites are also down-regulated by VH298 (Figure 4H). A greater correlation between hypoxia and VH298 accessibility changes is observed when comparing changes located at hypoxia up-regulated expression genes (Figure 4H). 53 hypoxia up-regulated expression genes (RNA-seq) display increased accessibility in response to hypoxia or VH298, sharing 16 gene regions, 22 unique to hypoxia treatment and 15 unique to VH298 treatment (Figure 4H).

This analysis establishes that VH298 partially mimics the hypoxia response, concerning loci specific increases in accessibility. Thus, HIF stabilisation, independent of oxygen sensing enzyme inhibition, is sufficient to drive a subset of hypoxia inducible changes in chromatin accessibility.

### Hypoxia and VH298 induced accessibility changes are also observed by ATAC-qPCR

**Figure 5.**
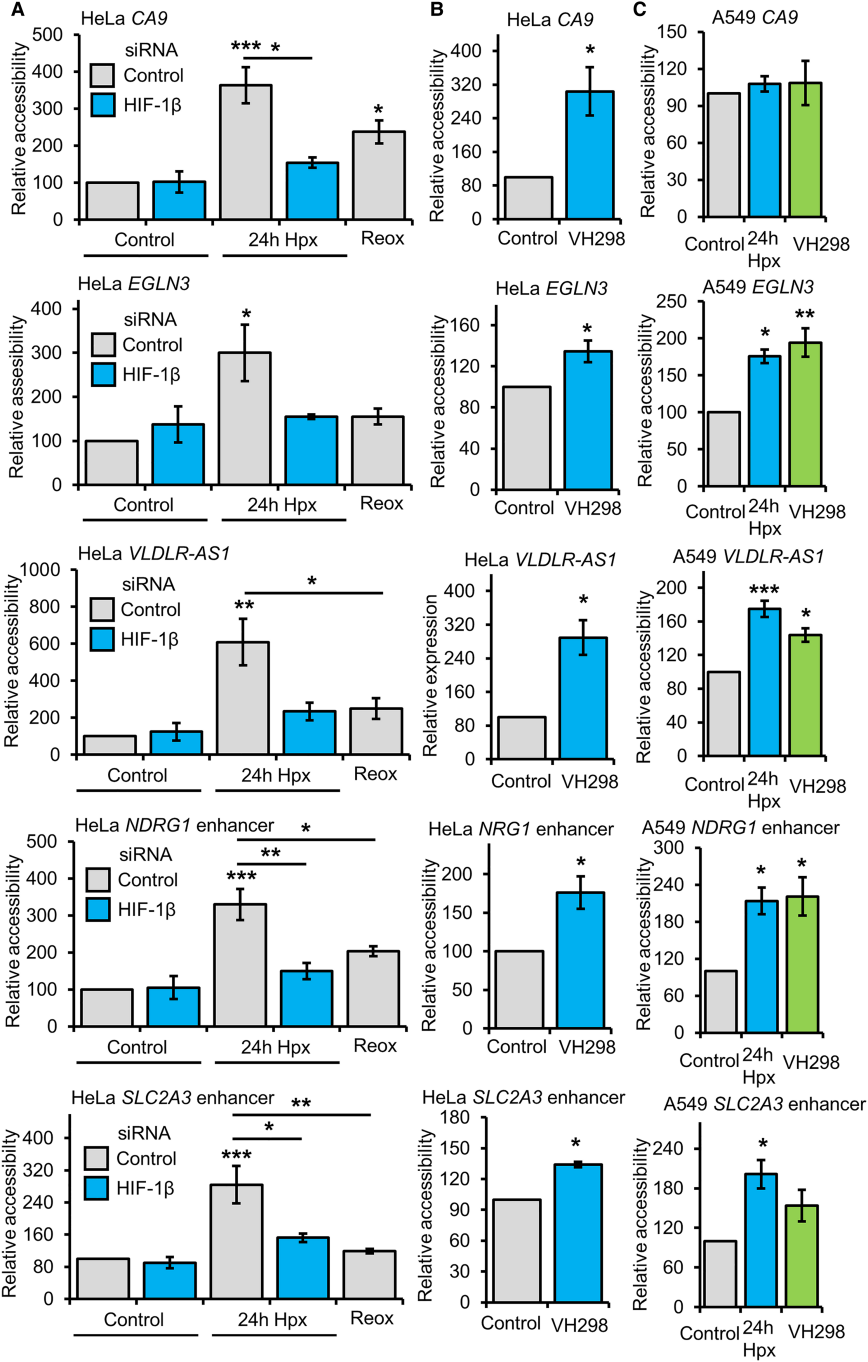
Validation of accessibility changes. (**A**) ATAC-qPCR analysis in HeLa cells cultured at 21% oxygen, transfected with control siRNA or HIF-1β siRNA, and exposed to 0 h (control), 24 h 1% oxygen (hypoxia (Hpx)) and 24 h hypoxia followed by 1 h at 21% oxygen (reoxygenation). (**B**) ATAC-qPCR analysis in HeLa cells cultured at 21% oxygen and treated with 24 h DMSO (control) and 24 h 100 µM VH298. (**C**) ATAC-qPCR analysis in A549 cells cultured at 21% oxygen with treated with 24 h DMSO (control), 24 h hypoxia and 24 h 100 µM VH298. Graphs show mean (*n* = 3) ± SEM, * *P* < 0.05, ** *P* < 0.01, *** *P* < 0.001. (**A**,**C**) Statistical significance was determined via one-way ANOVA with post-hoc Tukey test. (**B**) Statistical significance was determined via Student's *t*-test.

To confirm changes in chromatin accessibility in response to hypoxia and VH298 treatment, ATAC-qPCR analysis was performed on a set of loci identified by the ATAC-seq analysis (Figure 5). The *CA9* promoter displayed increased accessibility in response to 24 h hypoxia exposure and 24 h VH298 treatment, and this increase was reduced when HIF-1β is depleted in 24 h hypoxia exposed cells and when cells are reoxygenated following 24 h hypoxia exposure (Figure 5A,B). These results agree with the ATAC-seq analysis. Similar results were obtained for the *EGLN3* (protein name PHD3) gene body, *VLDLR AS-1* promoter, *NDRG1* enhancer and *SLC2A3* (protein name GLUT3) enhancer loci (Figure 5A,B). Also agreeing with the ATAC-seq analysis, promoter chromatin accessibility at the *FGF11* promoter was specifically increased in response to 24 h hypoxia but not 24 h VH298 treatment (Supplementary Figure S5A,B). To determine if these changes are also present in another human cancer cell line, we repeated 24 h hypoxia and VH298 treatment ATAC-qPCR analysis in A549 cells (Figure 5C; Supplementary Figure S5C). Increased accessibility in response to 24 h hypoxia and VH298 treatment at *EGLN3, VLDLR AS-1, NDRG1 and SLC2A3* was also found in A549 cells, although the increase in *SLC2A3* was not statistically significant (Figure 5C). No significant changes were present at the *CA9* promoter (Figure 5C). *FGF11* accessibility was also unaffected by VH298 treatment in both cell lines, and hypoxia up-regulated accessibility was only observed in HeLa cells (Supplementary Figure S5A). Absence of increased accessibility in response to hypoxia at *CA9* and *FGF11* loci in A549 cells is not explained by lack of transcript up-regulation, as both genes are up-regulated in response to 24 h hypoxia (determined by A549 RNA-seq (Supplementary Dataset S3). Differences in the presence of basal (normoxic (21% oxygen)) open chromatin regions between the 2 cell lines also does not account for the differences in chromatin accessibility responses at the *CA9* and *FGF11* promoters (Supplementary Dataset S8). Analysis of basal open chromatin regions identified from ATAC-seq data in HeLa and A549 cells finds the *CA9* hypoxia up-regulated accessibility region is open in basal conditions in both cell lines, and the *FGF11* hypoxia up-regulated accessibility region is not open in basal conditions in both cell lines (Supplementary Dataset S8). The cell type specific responses we observe could arise from differences in timing of chromatin changes in response to hypoxia or represent cell type heterogeneity in hypoxia inducible chromatin accessibility changes.

An open region of the *ACTB* promoter, which was unchanged in response to hypoxia and VH298 treatment in ATAC-seq analysis, was also analysed via ATAC-qPCR as a control (Supplementary Figure S5A–C). Immunoblotting for HIF-1α and HIF-2α was performed in A549 cells treated with 24 h hypoxia or 24 h, 100 µM VH298, confirming the expected hypoxia responsiveness/HIF-α isoform stabilisation in this cell line (Supplementary Figure S5D).

### Mechanistic insight into hypoxia inducible changes in chromatin accessibility

**Figure 6.**
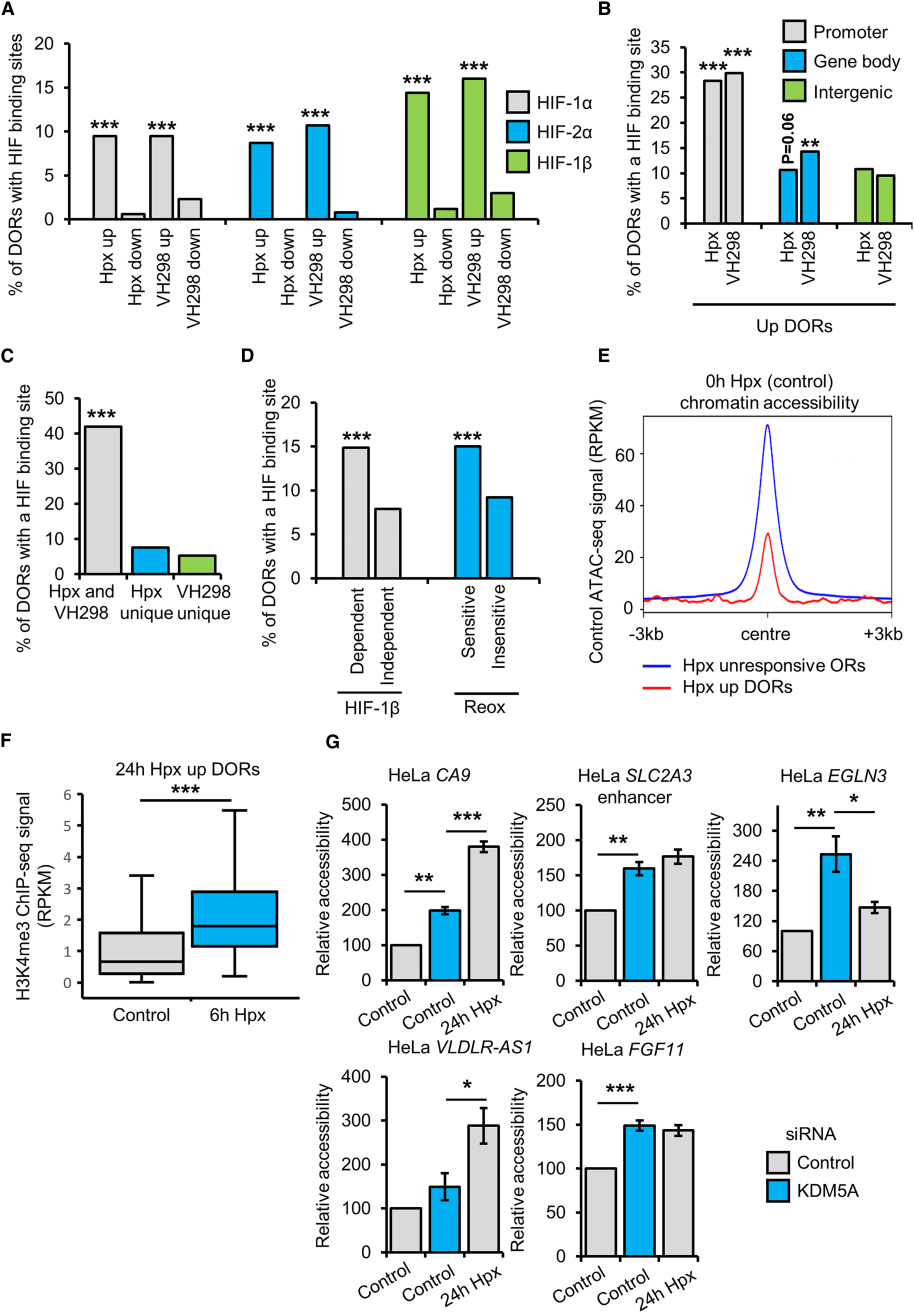
Mechanistic insight into hypoxia inducible changes in chromatin accessibility. (**A**–**D**) Overlap of differential open chromatin regions (DORs) identified by ATAC-seq (*n* = 2) with HIF subunit binding sites identified by ChIP-seq (*n* = 2) in HeLa cells. (**A**) Overlap of 24 h 1% oxygen (hypoxia (Hpx)) or 24 h, 100 µM VH298 DORs with of HIF subunit binding sites. Percentage of DORs containing a HIF binding site (HIF-1α, HIF-2α or HIF-1β) are displayed. (**B**) Overlap of 24 h hypoxia or VH298 up-regulated DORs with of HIF subunit binding sites. Percentage of promoter, gene body and intergenic DORs containing a HIF binding sites (HIF-1α, HIF-2α or HIF-1β) are displayed. (**C**) Overlap of both VH298 and 24 h hypoxia up-regulated DORs, 24 h hypoxia specific up-regulated DORs or VH298 specific up-regulated DORs with HIF subunit binding sites. Percentage of DORs containing a HIF binding site (HIF-1α, HIF2α or HIF1β) are displayed. (**D**) Overlap of HIF-1beta dependent/independent and reoxygenation sensitive/insensitive 24 h hypoxia up-regulated DORs with HIF subunit binding sites. Percentage of DORs containing a HIF binding site (HIF-1α, HIF-1β or HIF-2α) are displayed. (**A**–**D**) Statistical significance was determined via hypergeometric test, ** *P* < 0.01, *** *P* < 0.001. (**E**) Metagene plot of control condition (0 h hypoxia) ATAC-seq signal (RPKM) at the indicated regions. (**F**) Box plot of H3K4me3 ChIP-seq (*n* = 2) signal (RPKM) in HeLa cells exposed to 0 h (control) and 6 h hypoxia at 24 h hypoxia up-regulated DORs (centre ±1 kb). Statistical significance was determined via Wilcoxon signed-rank test, *** *P* < 0.001. (**G**) ATAC-qPCR analysis in HeLa cells cultured at 21% oxygen, transfected with control or KDM5A siRNA, and exposed to 0 h (control) or 24 h hypoxia. Graphs show mean (*n* = 3) ± SEM, statistical significance was determined via one-way ANOVA with post-hoc Tukey test, * *P* < 0.05, ** *P* < 0.01, *** *P* < 0.001.

To gain mechanistic insight into hypoxia/HIF driven changes in chromatin accessibility, we measured the percentage hypoxia and VH298 up-regulated DORs containing HIF isoform binding sites HeLa HIF ChIP-seq data (Figure 6A). HIF-1α, HIF-1β and HIF-2α binding sites are enriched at VH298 and 24 h hypoxia up-regulated DORs but not at down-regulated DORs (Figure 6A). This indicates a role of direct HIF binding in hypoxia/VH298 induced increases in chromatin accessibility complemented by HIF indirect changes. Next, we analysed HIF isoform binding sites at promoter, gene body and intergenic hypoxia or VH298 up-regulated DORs, finding HIF binding sites show the strongest preference for promoter DORs (Figure 6B; Supplementary Figure S6A). HIF binding sites are also more strongly enriched at DORs up-regulated in response to both hypoxia and VH298 compared with hypoxia unique and VH298 unique up-regulated DORs, suggesting that hypoxia unique and VH298 unique DORs may involve more HIF indirect changes (Figure 6C; Supplementary Figure S6B). Statistically significant overlaps of HIF isoform binding sites are present at reoxygenation sensitive and HIF-1β dependent hypoxia up-regulated DORs, but not at reoxygenation insensitive and HIF-1β independent hypoxia up-regulated DORs (Figure 6D; Supplementary Figure S6C). Thus, HIF binding is a determinant of reoxygenation sensitivity and HIF-1β dependence regarding hypoxia up-regulated DORs. As with hypoxia up-regulated DORs, HIF-1α and HIF-2α binding sites are similarly enriched at hypoxia up-regulated DORs located at hypoxia up-regulated genes (Supplementary Figure S6D). Analysis of hypoxia up-regulated DORs with HIF-α binding sites finds the majority display binding sites for both HIF-1α and HIF-2α (Supplementary Figure S6E,F). Therefore, differential HIF-α isoform occupancy does not seem to account for specificity of hypoxia induced up-regulation of chromatin accessibility.

Analysis of chromatin accessibility under in normoxia (21% oxygen) reveals hypoxia and VH298 induced increases in chromatin accessibility occur at both constitutively closed and open chromatin loci (Supplementary Figure S7A). Sixty-two percent of 24 h Hpx up-regulated DORs are open in normoxia (21% oxygen), as defined by presence of a high confidence (identified in all biological replicates, each with an FDR < 1 × 10^−15^) ORs in normoxia. Sixty-two percent of VH298 up-regulated DORs are open in normoxia and 72% of 24 h hypoxia up-regulated DORs at hypoxia up-regulated DEGs are open in normoxia (Supplementary Figure S7A). Mapping of basal (normoxic (21% oxygen)) chromatin accessibility signal to 24 h hypoxia up-regulated DORs and hypoxia unresponsive ORs shows lower normoxic chromatin accessibility at 24 h hypoxia up-regulated DORs (Figure 6E). Similar results are shown comparing normoxic chromatin accessibility at VH298 up-regulated DORs with VH298 unresponsive ORs (Supplementary Figure S7B) and comparing normoxic chromatin accessibility at 24 h hypoxia up-regulated DORs located on hypoxia up-regulated DEGs with hypoxia unresponsive ORs located on hypoxia up-regulated DEGs (Supplementary Figure S7C). This analysis indicates that up-regulation of chromatin accessibility in response to hypoxia or VH298 is preferred at loci with relatively low basal chromatin accessibility compared with unresponsive open chromatin regions. Basal gene expression has been shown to correlate with gene transcriptional responses to hypoxia, with genes transcriptional active under normal oxygen tensions being permissive for transcriptional regulation in hypoxia, and it is suggested that basal gene expression may define, in part, cell type specific responses to hypoxia [24]. Interestingly, unlike transcriptional responses, we find that basal gene expression does not appear to be a determinant of chromatin accessibility responsiveness in hypoxia, as there no correlation between basal gene expression and genes with chromatin accessibility responses (Supplementary Figure S7D–F). It will be important to establish if this is the case across other cell types.

To investigate a potential role of chromatin remodellers in hypoxia induced chromatin accessibility changes, we performed overlap analysis of VH298 and 24 h hypoxia DORs with publically available genome wide occupancy data in HeLa cells for members of the SWI/SNF complex (BRG1, BRG1-Associated Factor 155 (BAF155), BAF47, BAF170) and Sucrose Nonfermenting Protein 2 Homolog (SNF2H) (Supplementary Figure S8A). While there were no statistically significant overlaps, SWI/SNF binding sites were more prevalent at VH298 and 24 h hypoxia up-regulated DORs compared with down-regulated DORs. To test if SWI/SNF is required for hypoxia inducible increases in chromatin accessibility, we depleted BAF155, a core subunit of SWI/SNF complexes, with siRNA, and measured chromatin accessibility changes at the previously validated sites (Figure 5) by ATAC-qPCR (Supplementary Figure S8B). At all six hypoxia up-regulated DORs analysed, BAF155 depletion in hypoxia did not affect chromatin accessibility in hypoxia, demonstrating BAF155 is not required for hypoxia mediated increases in chromatin accessibility, at least the sites studied (Supplementary Figure S8B). An open region of the *ACTB* promoter, unaffected by hypoxia and VH298 treatment by ATAC-seq, was used as a control region (Supplementary Figure S8B). Immunoblot analysis confirmed effective depletion of BAF155 with siRNA treatment (Supplementary Figure S8C). As there is publically available HeLa cell ChIP-seq data for p300, a known HIF-α co-activator [25], we compared hypoxia/VH298 DORs with p300 binding sites (Supplementary Figure S8D). However, as with the chromatin remodeller overlaps, we found no statistically significant overrepresentation of p300 binding sites (Supplementary Figure S8D). A limitation of this analysis is that the p300 and chromatin remodeller data is in normoxic cells.

Changes to H3K4me3 in hypoxia correlate with changes in gene expression [13,26] and have been linked to coordination of hypoxia induced transcriptional changes [13]. Analysis of publically available H3K4me3 ChIP-seq data finds that in hypoxia, H3K4me3 is enriched at 24 h hypoxia up-regulated DORs (Figure 6F). As Lysine Demethylase 5A (KDM5A) depletion in normoxia has been shown to mimic hypoxia induced H3K4me3 and gene expression changes, we tested whether KDM5A depletion mimics hypoxia induced changes in chromatin accessibility (Figure 6G). ATAC-qPCR reveals that KDM5A depletion in normoxic HeLa cells increases chromatin accessibility at 4/5 hypoxia up-regulated DORs analysed (Figure 6G). An open region of the *ACTB* promoter, which was unchanged in response to hypoxia and VH298 treatment in ATAC-seq analysis, was also analysed via ATAC-qPCR as a control (Supplementary Figure S9A). Immunoblot analysis confirmed effective depletion of KDM5A with siRNA treatment (Supplementary Figure S9B). Taken together, these data suggest an intricate cross-talk between HIF and KDM5A in the control of hypoxia-induced chromatin structure changes.

## Discussion

ATAC-seq is utilised here to measure the chromatin accessibility landscape to response to hypoxia. Our findings that low oxygen triggers loci specific changes in chromatin accessibility agrees with previous ATAC-seq studies in other cell lines exposed to hypoxia [17,19,20]. We characterised genomic loci with differential accessibility in hypoxia, via integrative analysis with RNA-seq and publically available databases. This analysis reveals that changes in chromatin accessibility in hypoxia are associated with hypoxia responsive genes, both within genes and genes promoters, and at distal regulatory elements. Genes with differential accessibility include the core, well-characterised hypoxia responsive genes, including *CA9, EGLN3, SLC2A3* and *NDRG1*. Interestingly, the loci with one the biggest hypoxia induced change in accessibility is on *VLDLR-AS1*, an antisense transcript of the hypoxia inducible HIF target gene, *VLDLR* [27,28]. *VLDLR-AS1* gene expression was also elevated in hypoxia in our RNA-seq analysis in HeLa and A549 cells. This may represent a feedback loop to reduce *VLDLR* expression levels after prolonged exposure to hypoxia, similar to *HIF1A* and *HIF1A-AS2* [29]*.* However, further studies will be needed to confirm this hypothesis.

HIF is central to transcriptional responses in hypoxia [3–6]. Motif enrichment analysis identifies HIF subunit motifs are enrichment, specifically at sites with increased accessibility in hypoxia. By combining siRNA depletion of HIF-1β, the obligate dimerisation partner of HIF-1α and HIF-2α, in cells exposed to normal oxygen and low oxygen with ATAC-seq analysis, we determine the dependence of HIF on hypoxia induced chromatin accessibility variations. Most sites where shown to require HIF for hypoxia driven alterations to accessibility, with a stronger dependence for up-regulated accessibility loci compared with down-regulated accessibility loci. Many cellular responses to low oxygen are highly dynamic and reverted upon reoxygenation, including HIF pathway activation, due to oxygen sensing via oxygen dependent enzymes [2,9]. Our analysis also shows that the majority of accessibility changes in hypoxia are restored to normoxic levels following a short period of reoxygenation, demonstrating rapid and dynamic oxygen sensitivity, which parallels that of the HIF pathway.

Whilst the central oxygen-sensing pathway in metazoans is the VHL-PHD-HIF axis, impairment of other oxygen sensitive enzymes in low oxygen, and non-HIF targets PHD enzymes, can trigger other oxygen sensitive changes [15]. This includes changes to DNA and histone methylation [30]. In an attempt to delineate HIF stabilisation in hypoxia, from other effects caused by inhibition of cellular oxygen sensors, we performed ATAC-seq in cells treated with a chemical stabiliser of HIFα, called VH298. This compounds works via blocking the hydroxylated HIF-α binding pocket of VHL, thus stabilising both HIF-1α and HIF-2α and partially mimicking HIF mediated responses, without inhibiting oxygen sensors [21]. VH298 induces fewer changes in chromatin accessibility than hypoxia, and the changes induced by VH298 are also associated with hypoxia responsive genes and display HIF motif enrichment. 20% of hypoxia up-regulated chromatin accessibility sites are also increased in response to VH928. This analysis demonstrates that HIF-α isoform stabilisation is sufficient to trigger increases in chromatin accessibility, which partially mimic those driven by hypoxia. A similar trend was observed when previously elucidating proteome wide and transcriptome wide changes in response to hypoxia and VH298 [22,23]. There was strikingly little overlap between reduced accessibility loci in hypoxia and VH298. This is suggestive, that in hypoxia, additional oxygen dependent mechanisms impact on chromatin accessibility, acting in HIF dependent and independent loci. A limitation of the VH298 ATAC-seq experiment is we only use 1 time-point of VH298 and it is known that hypoxia and VH298 have different dynamics regarding HIF stabilisation and activation of HIF target genes [21–23]. Future work using multiple timpoints of VH298 and hypoxia will help distinguish changes in accessibility driven solely by HIF stabilisation and those which require additional oxygen sensing mechanisms.

ATAC-seq findings are confirmed with ATAC-qPCR validation at a subset of hypoxia inducible genes with increased chromatin accessibility. Repeated analysis in second human cancer cell line, A549, uncovers cell type specific responses, with 2 out the 6 sites studied (*CA9 and FGF11 promoters*) only displaying accessibility sensitivity to hypoxia in HeLa cells. Future investigation into chromatin responses across multiple cell types and hypoxia timepoints will help define cell conversed/specific responses as has been done previously for transcriptome response to hypoxia [31]. Current evidence points towards HIF isoform expression and activity, pre-established chromatin accessibility and local chromatin environment, including RNA pol2 availability, pre-existing promoter enhancer interactions at HREs, and HRE DNA methylation status, as key cell-type specificity determinants of hypoxia transcriptional responses [30]. Similar factors and additional chromatin regulation mechanisms may confer heterogeneity in chromatin accessibility responses across cell types, which may be linked to transcriptional responses. It has been suggested that basal gene expression profiles define, at least in part, cell type specific transitional responses to hypoxia, with genes transcriptionally active prior to hypoxia stimulation in a given cell type being susceptible to hypoxia induced transcriptional regulation [24]. Interestingly however, in our study, we find no correlation between basal gene expression and genes with chromatin accessibility responses to hypoxia, indicating basal gene expression is not important in determining which loci display hypoxia responsive changes in chromatin accessibility. While our analysis in HeLa cells would indicate otherwise, additional analysis across multiple cell types is required to establish if basal gene expression plays a role in selection of hypoxia responsive loci regarding chromatin accessibility.

Comparison to pan genomic HIF binding sites unveils strong enrichment of HIF-1α, HIF2-α and HIF-1β at loci with increased accessibility in response to hypoxia and VH298 treatment. HIF binding is favoured at promoter hypoxia inducible accessible sites over gene body and intergenic loci and is HIF is also a determinant of HIF dependence and reoxygenation sensitive of hypoxia inducible accessible sites. Taken together, these data support a model whereby hypoxia inducible increases in chromatin accessibility are mostly HIF dependent, and consist of both direct local HIF binding and HIF indirect mechanisms, with a small contribution from HIF independent mechanisms. Conversely, around half of the loci with hypoxia-repressed accessibility are HIF independent and most of the HIF dependent sites are regulated indirect of local HIF binding. Analysis of chromatin accessibility in normal oxygen tensions reveals genomic locations with increased accessibility in hypoxia have low normoxic accessibility relative to unresponsive open chromatin regions. Hypoxia induced increases in chromatin accessibility at loci linked to hypoxia up-regulated genes may be required to achieve full transcriptional activation at these genes in hypoxia. A limitation of this study is that it does not determine the relative contributions of HIF-1α and HIF-2α to hypoxia/HIF induced changes in chromatin accessibility. Analysis of HIF-α isoform genome occupancy data suggests differential HIF-α isoform occupancy does not account for specificity of hypoxia induced up-regulation of chromatin accessibility. Approaches depleting or inhibiting specific HIF-α isoforms and using cell lines lacking HIF-1α or HIF-2α are required to establish their roles in hypoxia induced chromatin accessibility regulation.

Chromatin remodellers regulate cellular responses to hypoxia [32–34].We conducted preliminary investigation into the role played by chromatin remodellers in hypoxia and VH298 mediated accessibility changes by correlating hypoxia and VH298 responsive loci with pan genomic binding sites for normoxic SWI/SNF members and SNF2H binding sites in HeLa. No statistically significant correlations occur with this analysis, although it is limited by lack chromatin remodeller pan genomic occupancy studies in hypoxia. There is greater proportion of SWI/SNF binding sites at hypoxia and VH298 up-regulated accessibility sites compared with down-regulated accessibility sites. However, ATAC-qPCR analysis finds that a core subunit of the SWI/SNF complex, BAF155, is not required for hypoxia induced accessibility changes at the set of loci we validated. However, we cannot rule out SWI/SNF involvement completely.

Histone methylation modifications are sensitive to hypoxia [30] and KDM5A is reported cellular oxygen sensor that regulates H3K4me3 in hypoxia [13]. We show that hypoxia induced up-regulation of H3K4me3 is enriched at hypoxia up-regulated accessibility sites. Furthermore, depletion KDM5A in normoxia, increases chromatin accessibility at some of validated hypoxia up-regulated genomic loci. Thus, KDM5A may play a part in hypoxic regulation of chromatin accessibility. The histone methyltransferase SET1B was recently found to function as a HIF-1α co-activator, which is also required for H3K4me3 changes in hypoxia [26]. As such, it will be important to elucidate the potential role of SET1B in hypoxia driven changes to the chromatin accessibility landscape. Histone acetylation and DNA methylation can influence chromatin accessibility, however these modifications and there effectors are not studied here.

Previous work has shown that HIF preferentially binds transcriptionally active loci under normal oxygen tensions [24], which contain pre-established open chromatin regions [35] and promoter enhancer loops [11], and HIF predominantly acts via release of pre-bound promoter-paused RNA pol2 [10,36,37]. Our work provides evidence for an additional mechanism of hypoxia/HIF in inducing alterations in chromatin accessibility at a small subset of hypoxia responsive genes and distal regulatory elements, which may help co-ordinate full transcriptional responses at these genes. Indeed, we provide evidence that KDM5A is required for changes in chromatin at specific sites in our cell system. Further studies are needed to elucidate potential contributions and mechanisms of chromatin modifying enzymes and establish if chromatin accessibility changes in hypoxia are required to co-ordinate transcriptional responses at the subgroup of genes displaying chromatin accessibility responses.

## Material and methods

### Cell culture

Human cervix carcinoma HeLa and human lung carcinoma A549 cell lines were obtained from the American Type Culture Collection (ATCC) (Manassas, VA, U.S.A.) and maintained in Dulbecco's modified Eagle's medium (DMEM) (Gibco/ThermoFisher, Paisley, U.K.) supplemented with 10% v/v foetal bovine serum (FBS) (Gibco/ThermoFisher, Paisley, U.K.), 2 mM L-glutamine (Lonza, Slough, U.K.), 100 units/ml penicillin (Lonza, Slough, U.K.) and 100 µg/ml streptomycin (Lonza, Slough, U.K.) at 5% CO_2_ and 37°C. Cell lines were cultures for no more than 30 passages and routinely tested for mycoplasma contamination using MycoAlert Mycoplasma Detection Kit (Lonza, Slough, U.K.).

### Treatments

Hypoxia treatments were performed by incubating cells in an InvivO_2_ 300 Hypoxia Workstation (Baker Ruskinn, Bridgend, Wales) at 1% O_2_, 5% CO_2_ and 37°C. Lysis of hypoxia treated cells was carried inside the hypoxia workstation to avoid reoxygenation. Reoxygenation treatments were performed by incubating cells for 24 h in hypoxia followed by 1 h incubation at 21% O_2_, 5% CO_2_ and 37°C. VHL binding to HIF-1/2α was inhibited by treating cells with 100 µM VH298 (Sigma, Gillingham, U.K.) for 24 h and DMSO was used as vehicle control (Sigma, Gillingham, U.K.).

### siRNA transfections

Cells were transfected with 27 nM of small interfering RNA (siRNA) oligonucleotides (Eurofins, Ebersberg, Germany) for 48 h using Interferin (Polyplus, Illkirch, France) transfection reagent according to manufacturer's instructions. The following siRNA were used: Control CAGUCGCGUUUGCGACUGG, HIF-1β GGUCAGCAGUCUUCCAUGA, KDM5A GAAGAAUUCUAGCCAUACA, BAF155, CUGUAUUCAUGUGAUUGAA.

### Immunoblotting

Cells were lysed in RIPA buffer (50 mM Tris–HCl, pH 8, 150 mM NaCl, 1% v/v NP-40, 0.25% w/v Na-deoxycholate, 0.1% w/v SDS, 10 mM NaF, 2 mM Na₃VO₄ and 1 tablet/10 ml, Complete, Mini, EDTA-free protease inhibitor (Roche, Welwyn Garden city, U.K.)). Samples were incubated for 10mins on ice, centrifuged at 13 000 rpm, 10 min and 4°C, and supernatants (RIPA soluble protein lysates) were collected. Standard SDS–PAGE and immunoblotting protocols were performed with 20 µg of protein per lane loaded on SDS–PAGE gels. The following primary antibodies were used for immunoblotting: HIF-1α (610958, BD Biosciences (Workingham, U.K.)), HIF-1β (3718, CST ((Leiden, Holland)), HIF-2α (7096, CST ((Leiden, Holland)), Actin (60009-1, Proteintech (Manchester, U.K.)), BAF155 (11956, CST ((Leiden, Holland)), KDM5A (3876, CST ((Leiden, Holland)). Three biological replicates were analysed per condition. Immunoblot figures are from one biological replicate, which is representative of all replicates.

### ATAC-seq

Assay for Transposase-Accessible Chromatin using sequencing (ATAC-seq) was performed using the following protocol adapted from [38,39]. Cells were washed directly on cell culture plates with in 2 ml DPBS (Gibco/ThermoFisher, Paisley, U.K.) and 1 ml of resuspension buffer (10 mM Tris–HCl, pH 7.5, 10 mM NaCl, 3 mM MgCl_2_). Lysis buffer (0.1% v/v NP-40, 0.1% v/v Tween-20, 0.1 mg/ml Digitonin ((Promega, Southampton, U.K.) in resuspension buffer) was added at a volume resulting in a cell concentration of 1000 cells/µl followed by gentle scraping with a cell scraper and transfer of the cell suspension to 1.5 ml Eppendorf tubes. Samples were incubated on ice for 3 min. An amount of 1 ml of wash buffer (0.1% v/v Tween-20 in resuspension buffer) was added and mixing was performed by inverting tubes three times. Samples were centrifuged at 1000***g***, 10 min and 4°C. The supernatant was discarded and the pellet (cell nuclei) was resuspended in 50 µl transposition mix (50% v/v 2× Tagment DNA (TD) Buffer (Illumina, Cambridge, U.K.), 32% v/v PBS, 0.5 µl final 0.1% v/v Tween-20, 0.1 mg/ml Digitonin (Promega, Southampton, U.K.), 5% v/v TDE1 Tagment DNA Enzyme (Illumina, Cambridge, U.K.) in nuclease free water (Sigma, Gillingham, U.K.)) by gentle pipetting up and down six times. Transposition (tagmentation) reaction was performed by incubating samples at 1000 rpm, 30 min and 37°C on a thermomixer. DNA was purified using the MinElute PCR Purification Kit (Qiagen, Manchester, U.K.) according to manufacturer's instructions, with DNA eluted in 10 µl Elution buffer from the kit. Tagmented DNA was amplified by PCR in the following reaction mix; 10 µl DNA, 10 µl nuclease free water (Sigma, Gillingham, U.K.), 2.5 µl of 25 µM forward primer (Nextera/Illumina i5 adaptors (Illumina, Cambridge, U.K.)), 2.5 µl of 25 µM reverse primer (Nextera/Illumina i7 adaptors (Illumina, Cambridge, U.K.)) and 25 µl NEBNext® Ultra™ II Q5 Master Mix (NEB, Hertfordshire, U.K.), with the following cycling conditions; 5 min 72°C, 30 s 98°C and 11 cycles of 10 s 98°C, 30 s 63°C and 1 min 72°C. Double-sided magnetic bead based DNA purification (to remove primer dimers and large >1000 bp fragments) was performed using Agencourt AMPure XP beads (Beckman Coulter, High Wycombe, U.K.). DNA was quality controlled using an Agilent 2100 Bioanalyzer (Agilent, Stockport, U.K.), multiplexed, size selected (170–650 bp) using a Pipin prep (Sage Science, Beverly, MA, U.S.A.) and sequenced using S1 chemistry (paired-end, 2 × 50 bp sequencing) on a Novaseq sequencer (Illumina, Cambridge, U.K.). Two biological replicates were analysed per condition.

### ATAC-seq data analysis

Reads in fastq files were trimmed for adaptors using Cutadapt and low quality score using Sickle. Reads were aligned to the human genome version hg38 (UCSC) using Bowtie2 [40], sorted and indexed binary alignment mapped (bam) files with mitochondrial reads removed were generated using Samtools [41]. Bam files were filtered to keep ‘only properly paired reads’ following ENCODE guidelines using Samtools [41]. PCR duplicates were removed from bam files using Picard. Number of reads in bam files and their fragment length distribution was determined using Samtools [41]. Open chromatin regions (ORs) for each biological replicate were identified using MACS2 [42] (–nomodel –shift -100 –extsize 200 -q 0.01) and filtered to remove ENCODE DAC hg38 blacklisted regions and regions with an FDR < 1 × 10^−15^ using GenomicRanges [43] and ChIPpeakAnno [44]. ORs for each replicate within a condition were overlapped using ChIPpeakAnno [44] and regions not present within the overlap were excluded. Library sized normalised (reads per kb per million reads (RPKM)) bigwig files and metagene graphs and heatmaps were made using deepTools [45]. Differential open chromatin regions (DORs) between two conditions were determined using DiffBind [46] (dba.count fragmentSize = 150), dba.normalise library = DBA_LIBSIZE_PEAKREADS, dba.analyze method = DBA_DESEQ2) with filtering for log_2_ fold change (>±0.58) and FDR (<0.05). A log_2_fold change >±0.58 cut-off is chosen to set a minimum value for the effect size whilst avoiding discarding potentially biologically relevant data points, that result from applying a higher cut-off. PCA plots were generated DiffBind [46]. Closest gene TSS to ORs/DORs and genomic annotation of ORs/DORs were identified using ChIPpeakAnno [44]. Overlap of DORs with each other or other genomic intervals was performed using ChIPpeakAnno [44]. Genomic annotations of DORs were assigned using ChIPseeker [47]. Promoters were defined as TSS ±3 kb, gene bodies were defined as regions more than 3 kb downstream of TSS and upstream of TES and all other regions were defined as intergenic. Genic regions were defined as promoter and gene body regions. Gene signature analysis was performed using the Molecular Signatures Database with hallmark gene sets [48,49]. Motif enrichment analysis was performed using HOMER with normoxic ORs set as background [50]. Gene set enrichment analysis, and enrichment analysis for normoxic gene expression was performed using WebGestalt [51]. Enhancer analysis was performed using the HACER database [52]. Volcano plots were made using R Bioconductor package EnhancedVolcano. Coverage tracks were produced using IGV [53].

### ATAC-qPCR

Pre-multiplexed ATAC-seq DNA was diluted to 0.5 ng/µl and qPCR analysis of chromatin accessibility was performed by running 3 µl of DNA on a Mx3005P qPCR platform (Stratagene/Agilent, Stockport, U.K.) with Brilliant II Sybr green reaction mix (Stratagene/Agilent, Stockport, U.K.) in a final reaction of 15 µls. The following qPCR primers were used; CA9 F CAGACAAACCTGTGAGACTTT and R TACGTGCATTGGAAACGAG, PHD3 F TACAGGGTGTTTGGGTTTG and R ACGTAGCCCTGTCACTC, FGF11 F CAGACAGACAGACAGACAGATG and R CGCTAGCTTGGCGAGAG, VLDR AS1 F CAGTCCCAGTGTGCATATTT and R CCTCTGGGTGTTAGCATTTC, ULK1 F GGTGGCCCTTCCTTCTTA and R GCTGGACAGAACCACTCT, ACTB F GCGGTGCTAGGAACTCAAA and R TACTCAGTGGACAGACCCAA, NDRG1 enhancer F AGAAGGTGTGCGTGTTTAG and R GATGACTCCAGAAACCAAGAG, GLUT3 enhancer F CTTAGTTGTATCTGGGTGTGG and R GAGAGGAGCAATGTCTGATG, ACTB F GCGGTGCTAGGAACTCAAA and R TACTCAGTGGACAGACCCAA. Three biological replicates were analysed per condition.

### RNA-seq

RNA was extracted from HeLa and A549 cells using an RNeasy Mini Kit (Qiagen, Manchester, U.K.). RNA was quality controlled using an Agilent 2100 Bioanalyzer (Agilent, Stockport, U.K.). Dual-indexed, strand specific RNA-seq libraries were generated using NEBNext polyA selection and Ultra Directional RNA library preparation kits (NEB, Hertfordshire, U.K.), multiplexed, and sequenced (Paired-end, 2 × 150 bp sequencing) on a HiSeq 4000 sequencer (Illumina, Cambridge, U.K.). Three biological replicates were analysed per condition.

### RNA-seq data analysis

Reads in fastq files were trimmed for adaptors using Cutadapt and low quality score using Sickle. Reads were aligned to the human genome version hg38 (GRCh38, Ensembl) using STAR [54] and the resulting binary alignment mapped (bam) files were indexed using Samtools [41]. Read counts for each transcript (GRCh38, Ensembl) were generated using Subread (featureCounts) [55]. Differential expression analysis were performed using R Bioconductor package DeSeq2 [56] with filtering for log_2_ fold change (>±0.58) and FDR (<0.05). A log_2_ fold change >±0.58 is chosen cut-off to set a minimum value for the effect size whilst avoiding discarding potentially biologically relevant data points, that result from applying a higher cut-off.

### Statistical analysis

For ATAC-qPCR analysis comparing two conditions, statistical significance was determined via Student's *t*-test. For ATAC-qPCR analysis comparing more than two conditions, statistical significance was determined via one-way ANOVA with post-hoc Tukey test. For overlap of genes with differentially accessible chromatin regions identified by ATAC-seq, with genes with differential RNA expression, statistical significance was determined via Fisher's exact test. For comparison of H3K4me3 ChIP signals, statistical significance was determined via Wilcoxon signed-rank test. For overlap of genomic regions, statistical significance was determined via hypergeometric test using makeVennDiagram function of using ChIPpeakAnno with default parameters [44]. For all other statistical analysis, default settings of the particular analysis tool were used. In all cases, * *P* < 0.05, ** *P* < 0.01, *** *P* < 0.001.

### Data mining of public available datasets

HeLa HIF and H3K4me3 ChIP-seq datasets [26] (GSE169040 and GSE159128) and HeLa ATAC-seq datasets [57,58] (GSE121840 and GSE106145) were downloaded from the Gene Expression Omnibus [1]. p300 (ENCFF631WOD), BRG1 (ENCFF216YDM), BAF47 (ENCFF572FHR), BAF155 (ENCFF492BST) and BAF170 (ENCFF253UAA) ChIP-seq datasets and A549 ATAC-seq dataset (ENCFF674RFR) were downloaded from the ENOCDE portal [59]. SNF2H ChIP-seq [60] (PRJEB8713) dataset was downloaded from the European Nucleotide Archive.

## Data Availability

ATAC-seq (GSE186342 and GSE186123) and RNA-seq (GSE186370) data are deposited at the Gene Expression Omnibus [1]. Source files for ATAC-qPCR data and immunoblot images are available upon request.

## Open Access

Open access for this article was enabled by the participation of University of Liverpool in an all-inclusive *Read & Publish* agreement with Portland Press and the Biochemical Society under a transformative agreement with JISC.

## Abbreviations

2-OGDDs: 2-OG dependent dioxygenases
DEGs: differentially expressed genes
DOR: differential open region
HIF: hypoxia inducible family
HREs: hypoxia response elements
ORs: open chromatin regions
PHD: Prolyl Hydroxylases
RPKM: reads per kb per million reads
VHL: von Hippel–Lindau
