# Supplementary material for: Regulation of chromatin accessibility by hypoxia and HIF
Source: Biochem J. 2022 Mar 28;479(6):767–86. doi: 10.1042/BCJ20220008 (PMC9022986; doi:10.1042/BCJ20220008)

Batie et al_Supplementary Information

Supplementary Figure Legends:

Supplementary Figure S1. Chromatin accessibility changes in response to hypoxia additional data. ATAC-seq (*n=2*) in HeLa cells cultured at 21% oxygen, transfected with control siRNA and exposed to 0h (control), 1h and 24h 1% oxygen (hypoxia (Hpx)). **A)** Overlap of open chromatin region (OR) genes. **B)** Overlap of ORs between different HeLa ATAC-seq (*n=2*) studies. **C)** Metagene plots of ATAC-seq signal (RPKM) at all gene promoters. **D)** Heatmap of ATAC-seq signal across all ORs and ranked by control high to low signal ORs. **E)** Number of low stringency (FDR <0.1) differentially open chromatin regions (DORs) and genes with DORs (DOR genes), and percentage relative to total ORs and OR genes. **F)** Number of upregulated and downregulated ORs and their genomic location.

Supplementary Figure S2. Motif enrichment analysis additional data. A) Motif enrichment analysis of control (0h 1% oxygen) open chromatin regions (ORs) from HeLa cell ATAC-seq (*n=2*) at 24h 1% oxygen (hypoxia) upregulated differentially expressed genes (DEGs) identified from RNA seq (*n=3*).

Supplementary Figure S3. Hypoxia inducible changes in open chromatin are mainly sensitive to reoxygenation HIF dependent additional data. HeLa cells were cultured in 21% oxygen; transfected with control siRNA or HIF-1β siRNA, and exposed to 0h (control), 1h, 24h 1% oxygen (hypoxia (Hpx)) and 24h hypoxia followed by 1h at 21% oxygen (reoxygenation (Reox)). **A, B)** Immunoblot of the indicated proteins (*n=3*). **C)** ATAC-seq *(n=2)* principal component analysis (PCA). **D)** Metagene plots of ATAC-seq signal (RPKM) at all gene promoters. **E)** Heatmap of ATAC-seq signal across all ORs and ranked by control high to low signal ORs.

Supplementary Figure S4. VH298 ATAC-seq additional data. HeLa cells were cultured at 21% oxygen and treated with 24h DMSO (control) and 100 µM VH298 for 24h. **A)** Immunoblot of the indicated proteins (*n=3*). **B)** ATAC-seq (*n=2*) overlap analysis of open chromatin regions (ORs) and OR genes. **C)** ATAC-seq analysis, number of high stringency (log2 fold change -/+0.58 and FDR <0.05) differentially open chromatin regions (DORs) and genes with DORs (DOR genes), and percentage relative to total ORs and OR genes. **D)** ATAC-seq analysis, number of upregulated and downregulated DORs and their genomic location. **E)** Metagene plots of ATAC-seq signal (RPKM) plotted indicated DORs and protein coding gene promoters. **F)** Heatmap of ATAC-seq signal, ranked by control high to low signal ORs. **G)** ATAC-seq analysis, number of low stringency (FDR <0.1) differentially open chromatin regions (DORs) and genes with DORs (DOR genes), and percentage relative to total ORs and OR genes. **H)** ATAC-seq analysis, number of upregulated and downregulated DORs (low stringency DORs) and their genomic location. **I)** ATAC-seq motif

enrichment analysis at VH298 upregulated DORs (high stringency), top 5 enriched motifs are displayed.

Supplementary Figure S5. Validation of accessibility changes additional data. A) ATAC-qPCR analysis in HeLa cells cultured at 21% oxygen, transfected with control siRNA or HIF-1β siRNA, and exposed to 0h (control), 24h 1% oxygen (hypoxia (Hpx)) and 24h hypoxia followed by 1h at 21% oxygen (reoxygenation). **B)** ATAC-qPCR analysis in HeLa cells cultured at 21% oxygen and treated with 24h DMSO (control) or 24h 100 µM VH298. **C)** ATAC-qPCR analysis in A549 cells cultured at 21% oxygen and with treated with 24h DMSO (control), 24h hypoxia and 24h 100 µM VH298. Graphs show mean ($n=3$) ± SEM. **A,C)** P values were calculated via one-way ANOVA with post-hoc Tukey test. **B)** P values were calculated via Student's t-test. **D)** Immunoblot of the indicated proteins in A549 cells cultured at 21% oxygen and with treated 24h hypoxia, 24h 100 µM VH298 and 24h DMSO ($n=3$).

Supplementary Figure S6. Additional HIF subunit binding analysis. Overlap of differential open chromatin regions (DORs) identified by ATAC-seq ($n=2$) with HIF subunit binding sites identified by ChIP-seq ($n=2$) in HeLa cells. **A)** Overlap of 24h 1% oxygen (hypoxia (Hpx)) or 24h, 100 µM VH298 upregulated DORs with of HIF subunit binding sites. Percentage of promoter, gene body and intergenic DORs containing HIF binding sites are displayed. **B)** Overlap of both VH298 and 24h hypoxia upregulated DORs, 24h hypoxia specific upregulated DORs or VH298 specific upregulated DORs with HIF subunit binding sites. Percentage of DORs containing HIF binding sites are displayed. **C)** Overlap of HIF-1β dependent and independent 24h hypoxia upregulated DORs, or reoxygenation sensitive and insensitive 24h hypoxia upregulated DORs, with HIF subunit binding sites. Percentage of DORs containing HIF binding sites are displayed. **D)** Overlap of 24h hypoxia upregulated DORs on 24h hypoxia upregulated differentially expressed genes (DEGs) with HIF subunit binding sites. Percentage of DORs containing HIF binding sites are displayed. **A-D)** Statistical significance was determined via hypergeometric test, $*P < 0.05$, $**P < 0.01$, $***P < 0.001$. **E)** Overlap of HIF-1α and HIF-2α binding sites at 24h hypoxia DORs containing a HIF-α binding site. **F)** Overlap of HIF-1α and HIF-2α binding sites at 24h hypoxia DORs on 24h hypoxia upregulated DEGs containing a HIF-α binding site.

Supplementary Figure S7. Analysis of basal chromatin accessibility and gene expression at loci with chromatin accessibility responses to hypoxia. ATAC-seq ($n=2$) in HeLa cells cultured at 21% oxygen, transfected with control siRNA and exposed to 0h (control) and 24h 1% oxygen (hypoxia (Hpx) or treated 24h DMSO (control) and 100 µM VH298 for 24h. **A)** Proportion of differential open regions (DORs) with open regions present or absent under control conditions. **B-C)** Metagene plots of control condition ATAC-seq signal (RPKM) at the indicated regions. **D)** Enrichment analysis of 24h hypoxia upregulated DOR genes against ranked gene list of control RNA expression (FPKM)

from all genes identified by RNA-seq (*n=3*) in the control condition. **E)** Enrichment analysis of 24h hypoxia upregulated DOR genes against ranked gene list of control RNA expression (FPKM) from all genes identified by RNA-seq in the control condition. **F)** Enrichment analysis of 24h hypoxia upregulated DEGs containing 24h hypoxia upregulated DORs, against ranked gene list of 24h hypoxia upregulated DEG control RNA expression (FPKM). **D-F)** For display purposes, genes with FPKM values of 0 or greater than 100 were excluded from the ranked gene list.

Supplementary Figure S8. Chromatin remodeller and p300 analysis. A) Overlap of 24h 1% oxygen (hypoxia (Hpx)) and 100 µM VH298 differentially open chromatin regions (DORs) identified by ATAC-seq (*n=2*) in HeLa cells with SWI/SNF subunit binding sites (BRG1, BAF47, BAF155 and BAF170) and SNF2H binding sites identified by ChIP-seq (*n=2*) in HeLa cells. Percentage of DORs containing a specified binding site are shown. **B)** ATAC-qPCR analysis in HeLa cells cultured at 21% oxygen, transfected with control or BAF155 siRNA, and exposed to 0h (control) or 24h hypoxia. Graphs show mean (*N3*) ± SEM, $*P < 0.05$, $**P < 0.01$, $***P < 0.001$. **C)** Immunoblot of the indicated proteins in HeLa cells transfected with control or BAF155 siRNA (*n=3*). **D)** Overlap of p300 binding sites identified by ChIP-seq (*n=2*) in HeLa cells with 24h hypoxia and VH298 upregulated DORs.

Supplementary Figure S9. Regulation of chromatin accessibility by KDMA additional data. A) ATAC-qPCR analysis in HeLa cells cultured at 21% oxygen, transfected with control or KDM5A siRNA, and exposed to 0h (control) or 24h hypoxia. Graphs show mean (*n=3*) ± SEM. **B)** Immunoblot analysis of the indicated proteins in HeLa cells transfected with control or KDM5A siRNA (*n=3*).

Supplementary Datasets:

Supplementary Dataset 1. ATAC-seq quality control and open chromatin regions (ORs).

Supplementary Dataset 2. ATAC-seq differential open chromatin region (DOR) analysis.

Supplementary Dataset 3. RNA-seq differential expressed genes (DEGs).

Supplementary Dataset 4. ATAC-seq and RNA-seq integrative analysis.

Supplementary Dataset 5. ATAC-seq HIF-1β independent differential open chromatin regions (DORs).

Supplementary Dataset 6. ATAC-seq reoxygenation sensitive/insensitive differential open chromatin regions (DORs).

Supplementary Dataset 7. Hypoxia and VH298 ATAC-seq differential open chromatin regions (DORs) overlap analysis.

Supplementary Dataset 8. ATAC-seq open chromatin regions (ORs) in A549 cells.

Supplementary Figure S1.

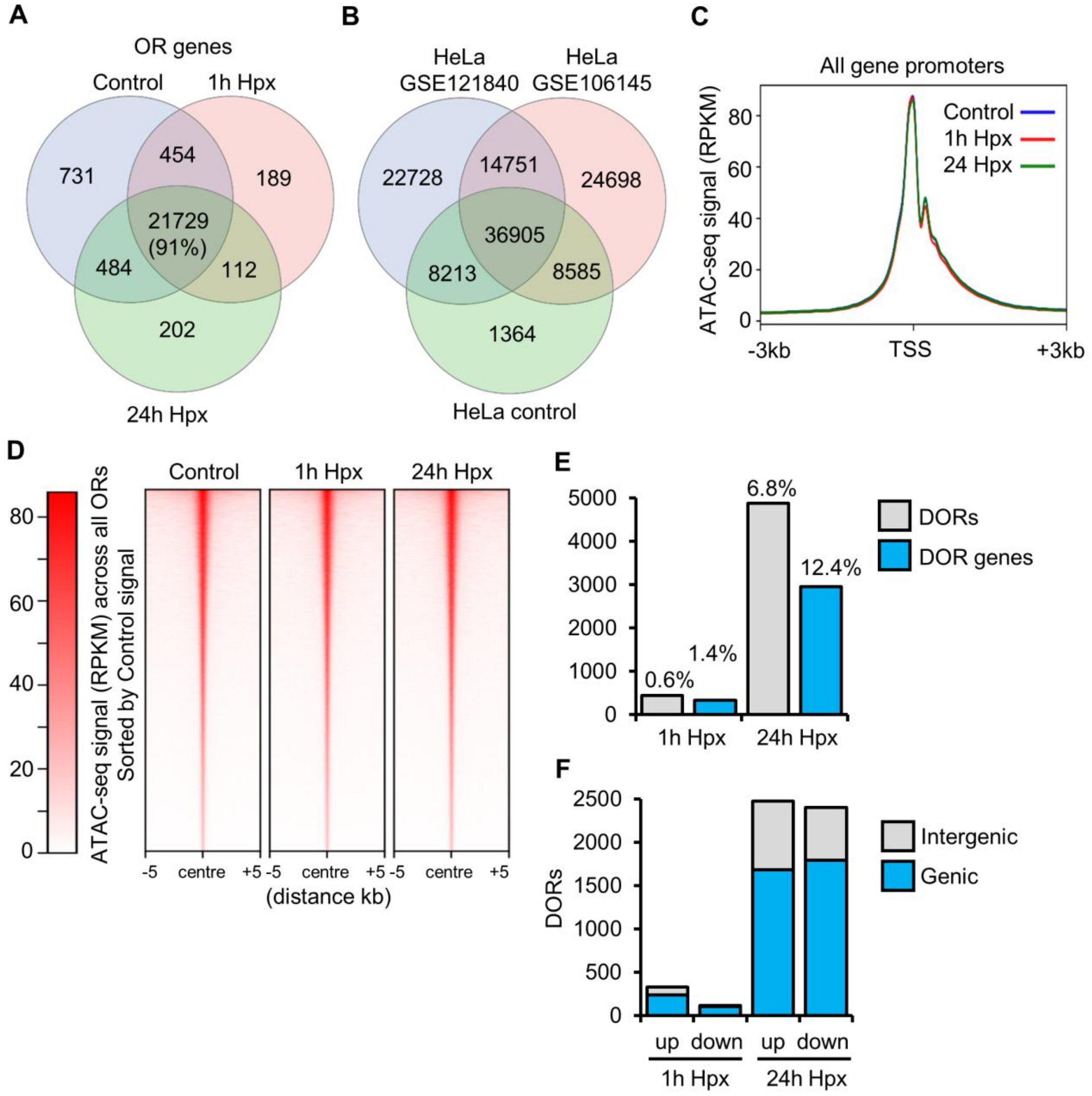

Supplementary Figure S2.

A

Motif analysis for 24h Hpx up DEGs

Motif	Protein	p value
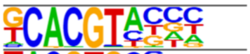	HIF-2α	$1X10^{-13}$
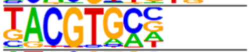	HIF-1α	$1X10^{-8}$
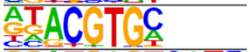	HIF-1β	$1X10^{-5}$
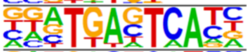	FOS	$1X10^{-5}$
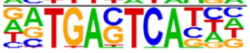	ATF3	$1X10^{-4}$

Supplementary Figure S3.

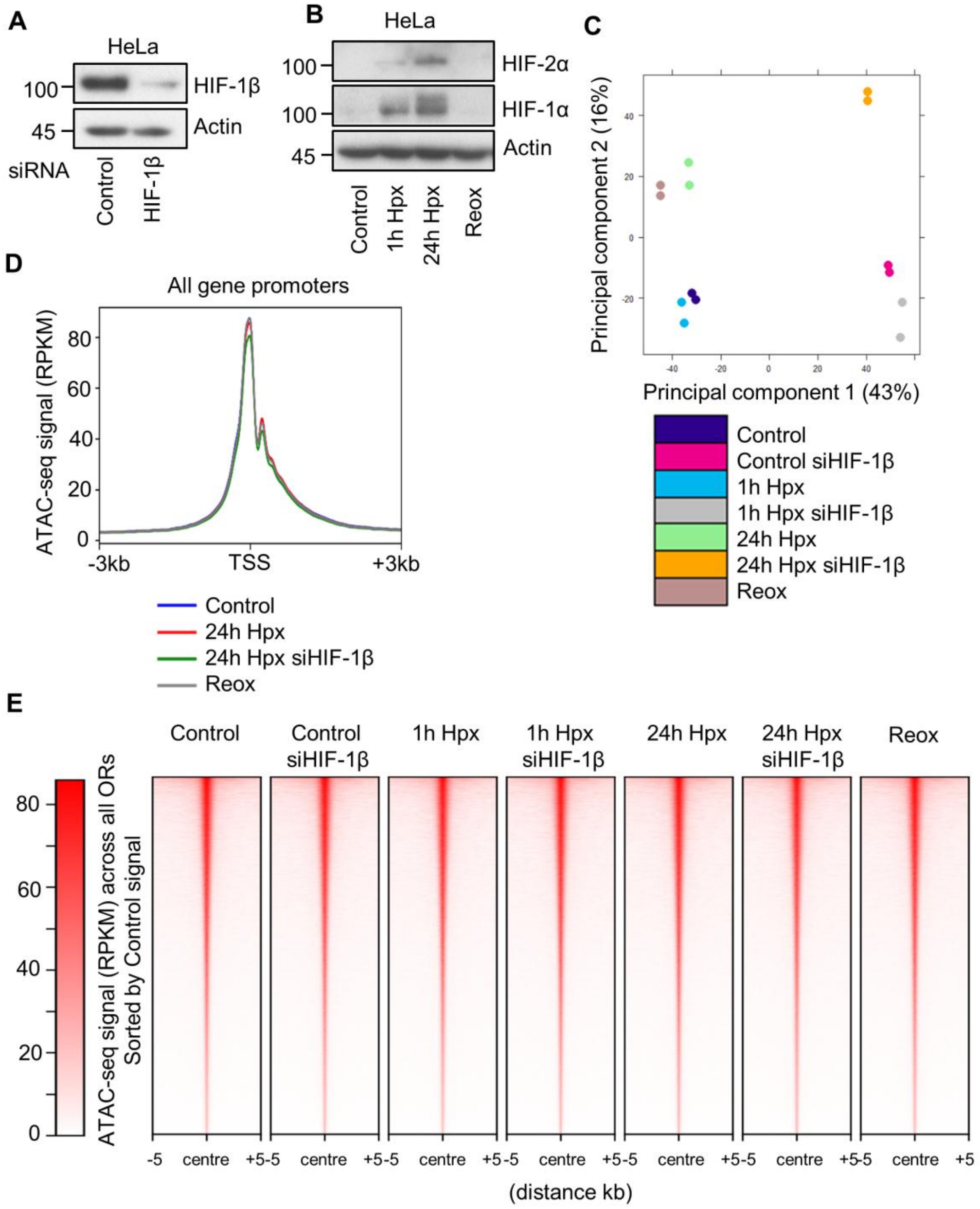

Supplementary Figure S4.

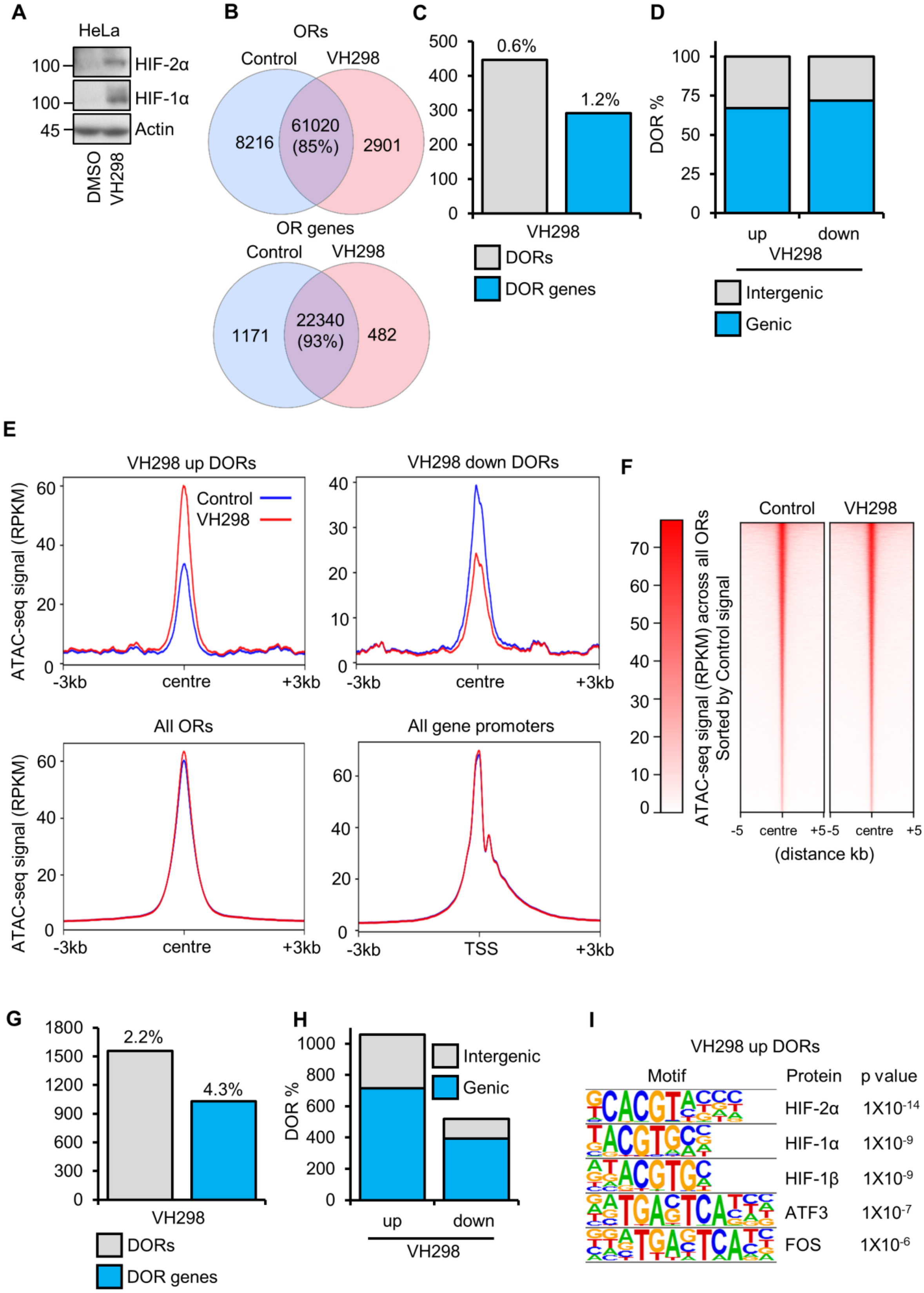

Supplementary Figure S5.

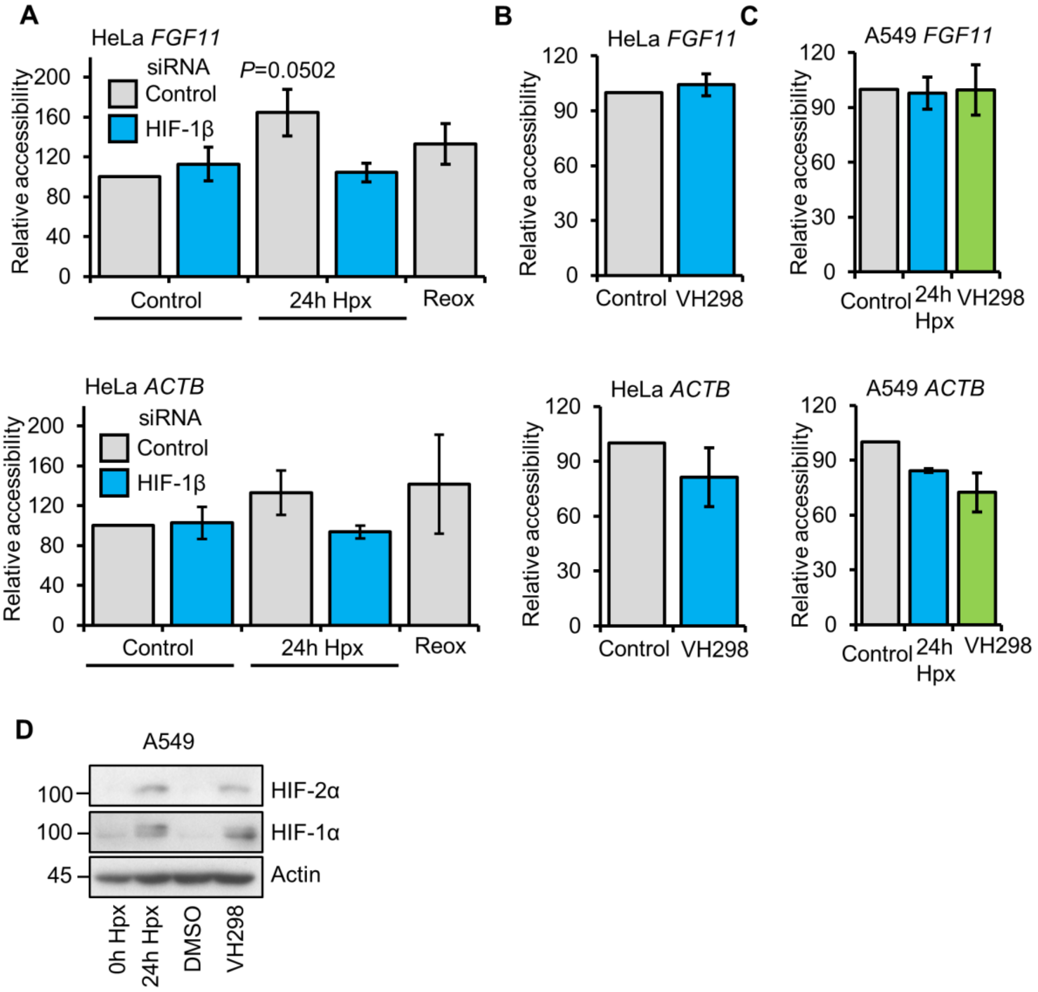

Supplementary Figure S6.

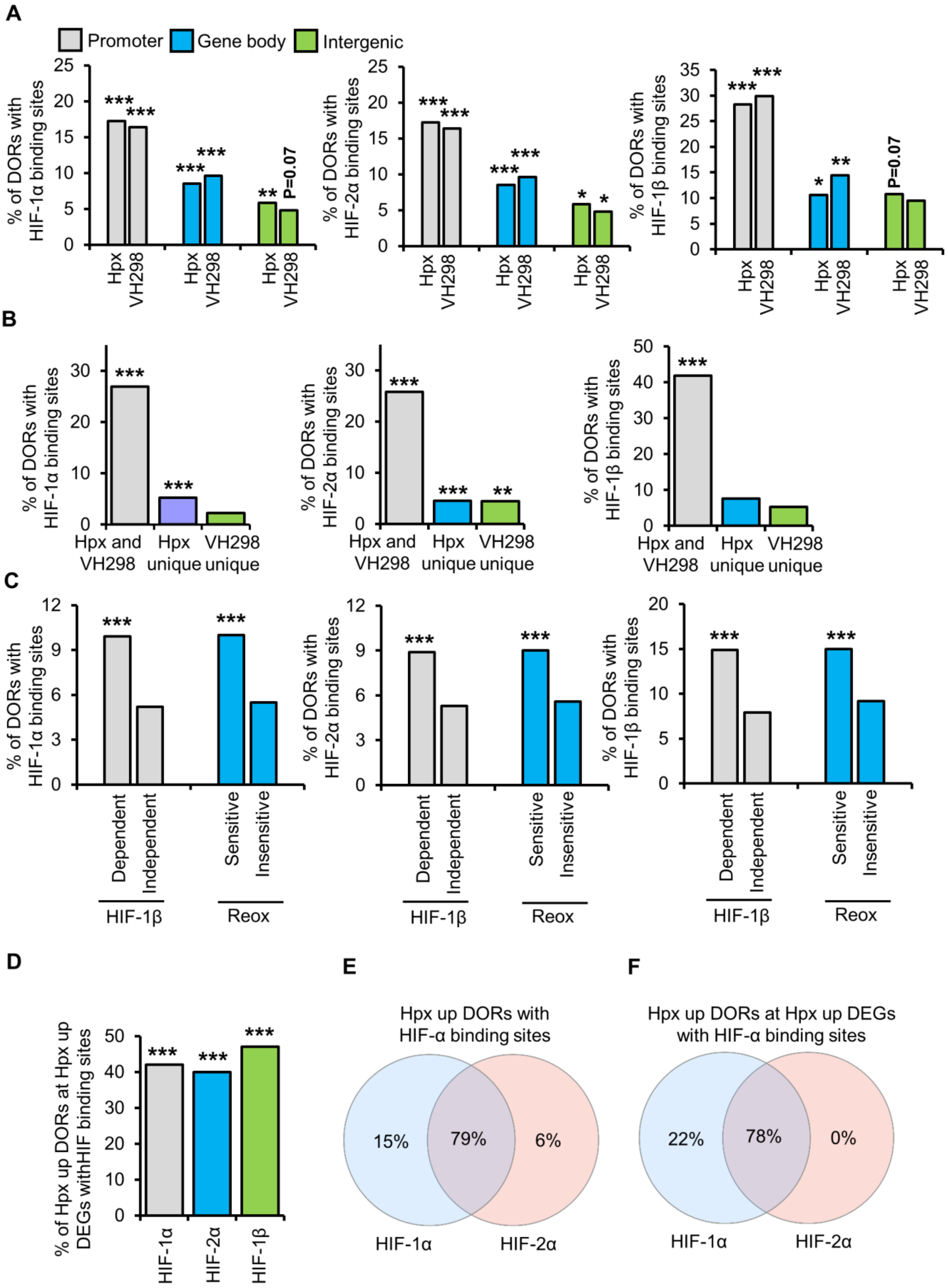

Supplementary Figure S7.

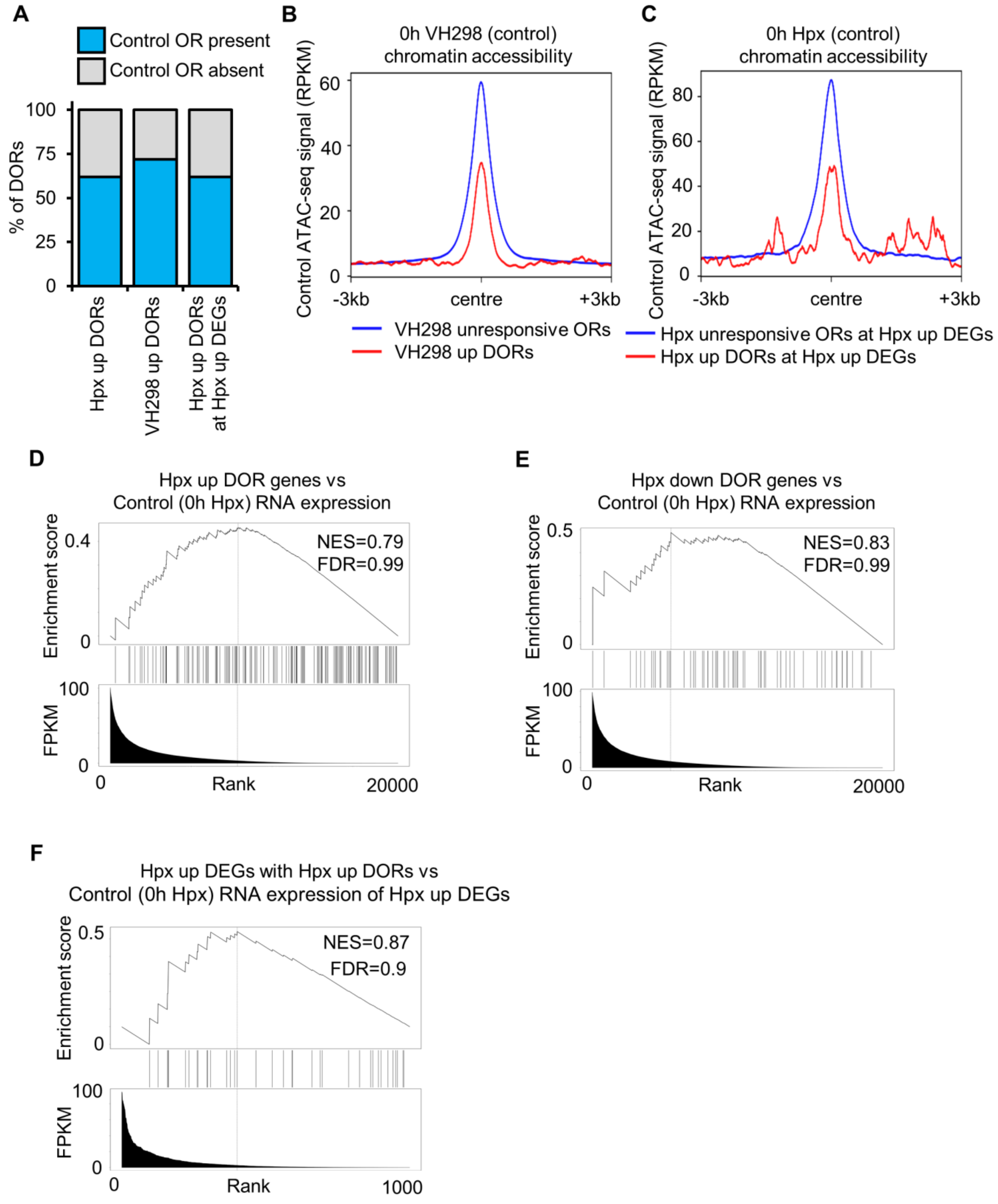

Supplementary Figure S8.

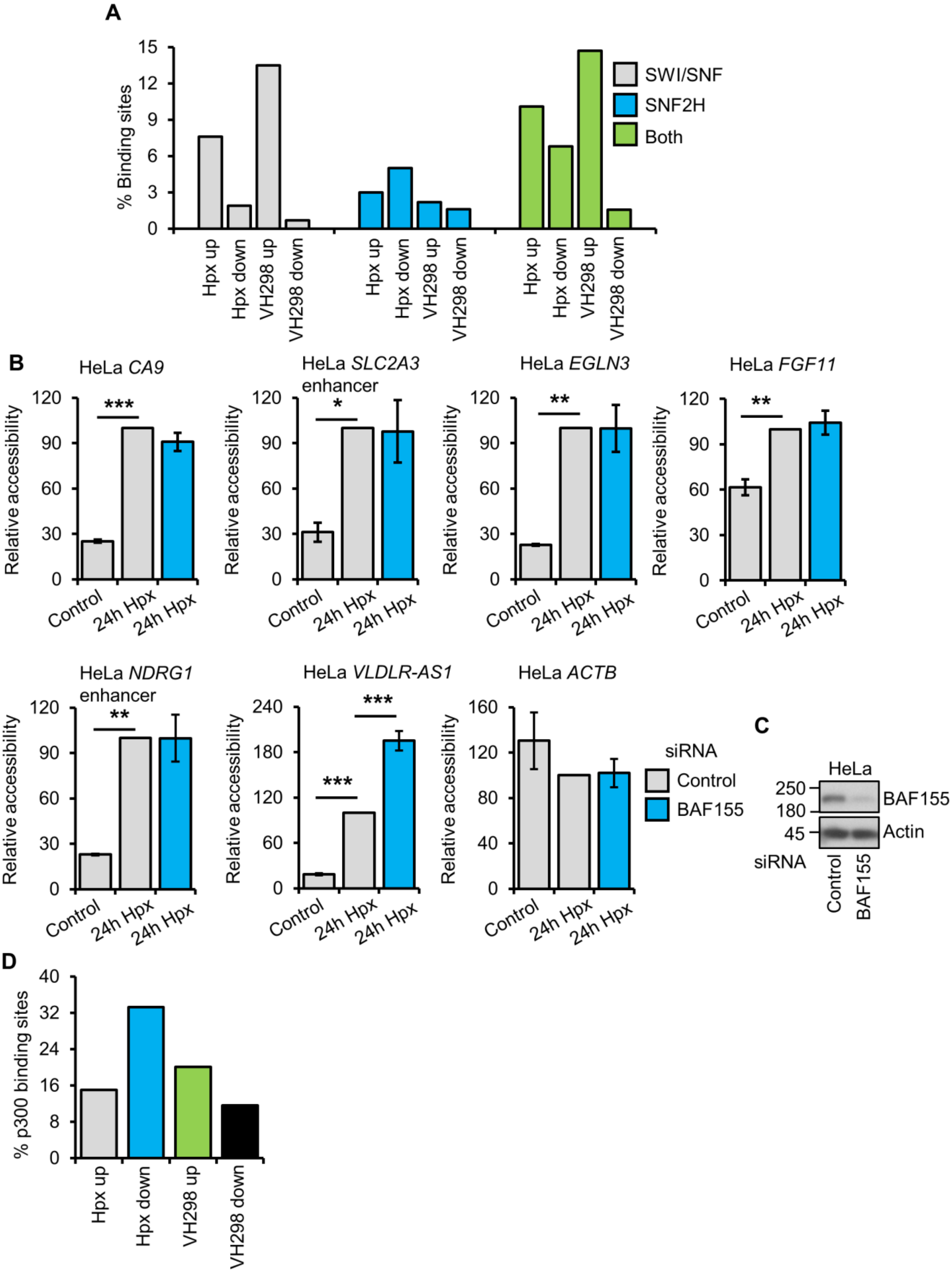

Supplementary Figure S9.

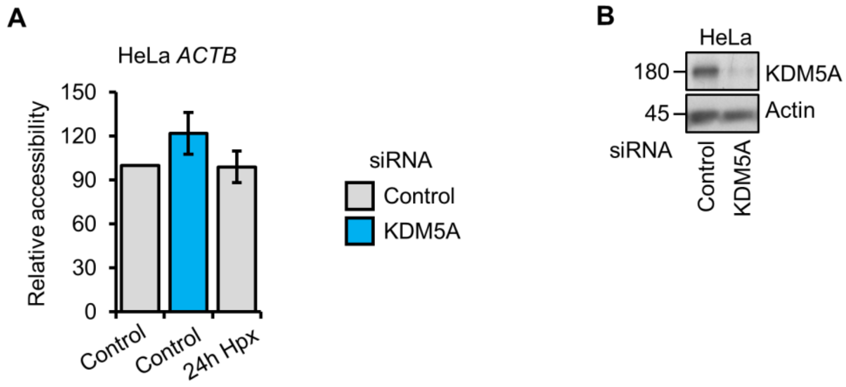

Supplement: Supplementary Material 1 [file BCJ-479-767-s1.pdf]
